# SpiNNTools: The Execution Engine for the SpiNNaker Platform

**DOI:** 10.3389/fnins.2019.00231

**Published:** 2019-03-26

**Authors:** Andrew G. D. Rowley, Christian Brenninkmeijer, Simon Davidson, Donal Fellows, Andrew Gait, David R. Lester, Luis A. Plana, Oliver Rhodes, Alan B. Stokes, Steve B. Furber

**Affiliations:** Advanced Processor Technologies Group, School of Computer Science, University of Manchester, Manchester, United Kingdom

**Keywords:** neuromorphic, SpiNNaker machine, framework, software, multiprocessing, parallel, middleware

## Abstract

SpiNNaker is a massively parallel distributed architecture primarily focused on real time simulation of spiking neural networks. The largest realization of the architecture consists of one million general purpose processors, making it the largest neuromorphic computing platform in the world at the present time. Utilizing these processors efficiently requires expert knowledge of the architecture to generate executable code and to harness the potential of the unique inter-processor communications infra-structure that lies at the heart of the SpiNNaker architecture. This work introduces a software suite called *SpiNNTools* that can map a computational problem described as a graph into the required set of executables, application data and routing information necessary for simulation on this novel machine. The SpiNNaker architecture is highly scalable, giving rise to unique challenges in mapping the problem to the machines resources, loading the generated files to the machine and subsequently retrieving the results of simulation. In this paper we describe these challenges in detail and the solutions implemented.

## 1. Introduction

With Moore's Law (Moore, 1965) coming to an end, the use of parallelism is now the principle means of continuing the relentless drive toward more and more computing power, leading to a proliferation of distributed and parallel computing platforms. These range from computing clusters such as Amazon Web Services (Murty, 2008) and the high throughput Condor platform (Thain et al., 2005), through to crowd sourcing techniques, such as BOINC (Anderson, 2004). Utilizing these types of resources often requires expert, platform-specific knowledge to create and debug code that is designed to be executed in a distributed and parallel fashion. In some cases, software stacks have been created that try to abstract this process away from the end user by the use of explicit interfaces (Message Passing Interface Forum, 1994; Dagum and Menon, 1998), or to re-cast the problem in a form that is easier to map into a distributed system (Dean and Ghemawat, 2008).

A SpiNNaker machine (Furber et al., 2013) is one such distributed parallel computing platform; SpiNNaker is a highly scalable low-power architecture whose primary application is the simulation of massively-parallel spiking neural networks in real time. Focusing on energy efficiency and the minimization of power-hungry data transfer between chips, SpiNNaker uses low performance off-the-shelf ARM processors as its basic computing elements coupled with a simple packet routing fabric to communicate across large arrays of individual SpiNNaker chips in a fraction of a millisecond. Each chip uses up to 1W when all the processors are fully utilized. To save energy, chips and even entire boards can be turned off when not in use. A growing number of users are now using SpiNNaker for various tasks, including Computational Neuroscience (Albada et al., 2018) and Neuro-robotics (Denk et al., 2013; Adams et al., 2014; Richter et al., 2016) for which the platform was originally designed, but also machine learning (Stromatias et al., 2015), and general parallel computation tasks, such as Markov chain Monte Carlo simulations (Mendat et al., 2015). The provision of a software stack for this platform aims to provide a base for the various applications, making it easier for them to exploit the full potential of the platform. Using standard, well-documented APIs internally, also allows users a smooth upgrade path to access ongoing improvements in the underlying tools without requiring changes to their software (or at most only minor changes should any interface changes be demanded).

The SpiNNTools software stack described in this work is currently released as part of the sPyNNaker software stack (Rowley et al., 2017b), but can be used fully without using sPyNNaker itself^1^. A thin layer which simplifies some of the interaction, known as the SpiNNakerGraphFrontEnd (Rowley et al., 2017a), has also been released as a Python library and is available at https://pypi.org/project/SpiNNakerGraphFrontEnd/1!4.0.0/.

SpiNNTools allows the user to describe their computational requirements in the form of a graph, where the vertices represent the units of computation and the edges represent allowed pathways of communication of data between the computational units. The graph is described in a high level language and the software then maps this directly onto an available SpiNNaker machine.

This paper describes the functionality of the software stack as of version 4.0.0 of sPyNNaker (Rowley et al., 2017b) and version 4.0.0 of SpiNNakerGraphFrontEnd (Rowley et al., 2017a) and is structured as follows. Section 2 describes the SpiNNaker architecture in more detail, explaining the machine onto which problems are mapped. We then discuss the software with which the SpiNNaker application cores are programmed in section 4. For context, in section 3 we review previous tool chains for SpiNNaker that have tried to solve the same problem in the past. This is followed by a discussion of the data structures required in section 5. We then go in to the details of how these structures are used to map the graph on to the machine in section 6, followed by an evaluation of the tools on some example applications that can be described as a graph in section 7. We outline further work to be done in section 8 and conclude in section 9.

## 2. SpiNNaker Architecture

**Figure 1:**
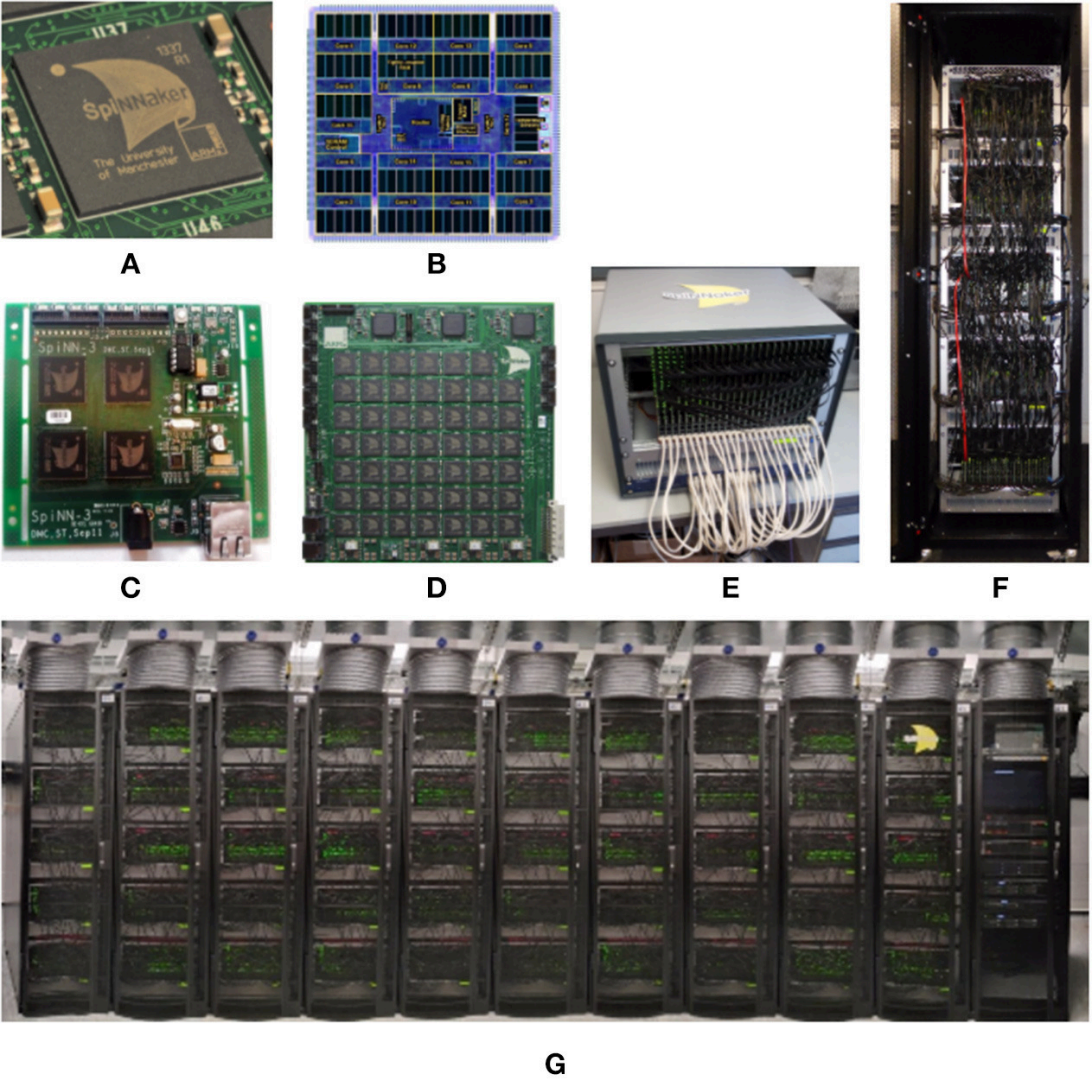
SpiNNaker Chip, Boards, and Machines. The SpiNNTools software works with single boards and multi-board machines. Single boards can be used in isolation; multi-board machines are wired into a torus, but can also be split into smaller multi- or single-board machines. **(A)** The SpiNNaker chip in packaging. **(B)** The SpiNNaker chip in detail. **(C)** A 4-chip SpiNN-3 board. **(D)** A 48-chip SpiNN-5 board. **(E)** A card frame made up of 24 SpiNN-5 boards wired together as a single machine. **(F)** A cabinet containing 5 card frames seen in **(E)** with a total of 120 SpiNN-5 boards wired together as a single machine. **(G)** 10 cabinets as seen in **(F)** with a total of 1,200 SpiNN-5 boards wired together as a single machine. This machine has over 1-million cores when operated as a single machine.

In this section we will look at the SpiNNaker Architecture, reviewing the resources available on each SpiNNaker node and the mechanisms that are available to manage the flow of data across a SpiNNaker machine. Each such machine is constructed from one or more SpiNNaker boards that are themselves made up of a number of SpiNNaker chips. There are two production versions of the SpiNNaker board, known as SpiNN-3 and SpiNN-5 which have 4 and 48 chips, respectively; the latter additionally has 3 FPGAs to allow it to be connected to up to 6 other boards to make up a larger SpiNNaker machine. Common machine configurations are shown in Figure 1. From a programming perspective the two board types and the machine types are identical, in that they consist of an array of connected SpiNNaker chips.

Figure 1B shows a graphical representation of a SpiNNaker chip, containing:

- 18 ARM968E-S (ARM, 2004) processors (also referred to as cores), operating at 200MHz, each of which has:
- 32 KiB of core-local, tightly-coupled instruction memory, referred to as ITCM, into which the entire processor scoped application code must fit
- 64 KiB of core-local, tightly-coupled data memory, referred to as DTCM, into which all locally scoped data and the application stack must fit
- a Direct Memory Access (DMA) controller for transferring data between the core-local and node-local memory
- an SDRAM controller giving access to 128 MiB of node-local SDRAM (not shown in the diagram as it is wire-bonded onto the chip) which can store large data structures, at the penalty of increased access latency relative to access time for data stored in local memory
- the SpiNNaker packet router, support packet routing communications (described in detail later)
- on-board sensors for hardware monitoring

**Figure 2:**
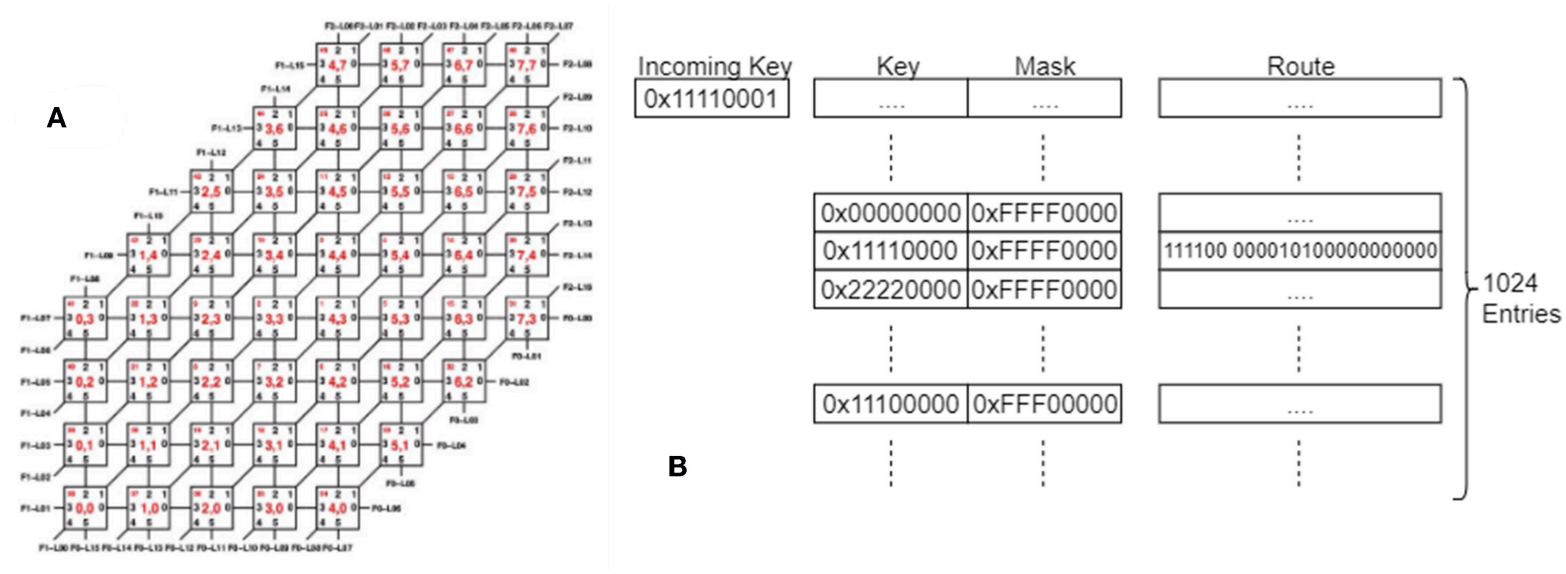
**(A)** The SpiNNaker Board wiring, showing the links between the chips on a single board, and the numbering of the links and chips. **(B)** The SpiNNaker Routing tables. An incoming key is matched against the entries in the table using the mask to determine which bits can be ignored and a route is determined, made up of 6 link bits and 18 processor bits, indicating where the packet should be sent. An entry might match with multiple keys once masked, in which case the match that appears earliest in the table is used.

The SpiNNaker router on each chip has six links with which it sends and receives SpiNNaker packets (Furber et al., 2013) from its neighboring chips. When arranged in a standard Cartesian 2D plane with the x-axis being labeled East/West and the y-axis being labeled North/South, the connections are usually made to chips in the directions of North, North East, East, South, South West, and West. The router can also send and receive SpiNNaker packets to and from any of the processors running on its own chip. The wiring of an individual SpiNN-5 board is shown in Figure 2A.

Data sent by an application makes use of the *multicast routing*, meaning that a packet sent from a single source core can be routed to multiple destinations simultaneously—useful functionality when implementing a neural network in which each neuron typically has a large fan-out. Each multicast *packet* consists of a key and an optional payload. A packet is generated by a core and is dropped into the communications fabric (and can then be forgotten about by the sender). It is then the responsibility of the hardware to ensure that the packet is routed to its destination(s), passing from chip to chip via the inter-chip links. Since it carries only a 32-bit key, there is not enough information in each packet alone to decide how to route it. This information is distributed across the network of SpiNNaker nodes, in the routing table stored within the Router of each chip. These tables must be set up with an ordered list of up to 1,024 entries, each of which has a key, a mask, and a route, as shown in Figure 2B. When a packet is received whose key matches the key of a (masked) entry, the associated *route* field is consulted. The route is a vector of bits, one per core on the same chip (18 in total) and one per external link (6 in total). If the bit is set, a copy of the packet is sent to that destination. In this way, a single incoming packet can spawn up to 24 copies of itself at each node in the network. Note that the use of a mask in each entry allows groups of incoming keys with overlapping (but non-identical) values to be routed using a single entry in the routing table; if the result of the bit-wise AND operation between the received key and the mask in the table matches the key in the table on the same row, this is considered a match on that entry. The list of entries in the routing table is ordered so that the first match is taken over any other. If no match is found, the packet is routed out of the opposite link to the one on which it was received (called *default routing*); thus packets will travel in a straight line through chips if not redirected by a routing entry. If the packet was received from a local processor, and no entry matches, the packet is dropped.

Due to the asynchronous nature of the SpiNNaker machine's communication fabric, it is possible to cause deadlocks within the system by having loops of communication. To avoid the traffic coming to a standstill, the SpiNNaker router can drop the next packet to be sent after a given time period (see Furber et al., 2013 for a more detailed explanation). If a packet is dropped, an interrupt is raised so that it can be detected and potentially dealt with (see later).

Each chip additionally has an Ethernet controller, although in practice only one chip is connected to the Ethernet connector on each board. The chip with the Ethernet connected to it is then called the *Ethernet chip*, and is used to communicate with the outside world, allowing, for example, the loading of data and applications. Communications with other chips on a board from outside of the machine must therefore go via the Ethernet chip; system-level packets are used to effect the communication between chips. In practice, the Ethernet connector of every board in a SpiNNaker machine is connected and configured, though it isn't a requirement to operation since every Ethernet chip can communicate with every other chip in the machine.

SpiNNaker machines are designed to be fault tolerant, so it is possible to have a functional machine with some missing parts. For example, it is normal that some of the SpiNNaker chips have 17 instead of 18 working cores, and sometimes even less as operational cores are tested more thoroughly than the testing done at manufacture. Additionally, machines can have whole chips that have been found to have faults, as well as some links missing between the chips and boards. The machine includes memory on to which faults can be stored statically in a *blacklist*, so that during the boot process these parts of the machine can be hidden to avoid using them.

SpiNNaker machines can be connected to external devices through either a *SpiNNaker-Link* connector, of which there is one on every 48-node board, or a *SpiNN-Link* SATA connector, of which there are 9 on each board; of those, 6 are used to connect to other boards. This, along with the low power requirements, make the machine particularly useful for robotics applications, since the board can be connected directly to the robot without any need of other equipment. The only requirement is that the external devices must be configured to talk to the machine using SpiNNaker packets. The links can be configured to directly connect with the links connected to a subset of the SpiNNaker chips on the board, and so entries in the routing tables of the chip can be used to send packets to any connected device and similarly to route packets received from the devices across the SpiNNaker network.

Some of the key design decisions made in developing the SpiNNaker architecture have important implications for the software which can run on the system: it must be possible to break up the computation of the application into units small enough that the code for each part fits on a single core, since cores cannot access code space outside of its own local memory. In contrast, the SDRAM is shared between the cores on a single chip, allowing cores to operate on the same data within the same chip. Data can be passed between chips only via the multicast routing mechanism. The implications of these design choices will be described in the remainder of the document.

## 3. Previous Software Versions

Software for SpiNNaker has been released in a software package known as *PACMAN48* (Galluppi et al., 2012). This software requires end users to load compiled applications and routing tables manually onto the SpiNNaker machine through the use of a low level debug tool developed in-house called *ybug*^2^. Other parts of the package are designed to ease the development of application code for end users. These consist of:

- low level libraries *SARK* and *Spin1API* running on each core of a SpiNNaker machine (described in more detail in the next section)
- an executable called *SCAMP* to run on one core of each chip, designating it as supervisor for that chip (described in the next section)
- a collection of C code which represented models known in the neuroscience community and defined by the PyNN 0.6 language (Davison et al., 2008)
- a collection of Python code which translates PyNN models onto a SpiNNaker machine

The PACMAN48 software has the following limitations:

- It only supports SpiNNaker machines consisting of a single SpiNN-3 or SpiNN-5 board
- It was designed only to support the computational neuroscience community, and thus non-neural applications are not supported
- End users are still expected to have expertise in using the SpiNNaker hardware, as they are expected to manually run separate scripts which together:
  - Boot the SpiNNaker machine
  - Load executables onto the SpiNNaker machine
  - Load data objects onto SpiNNaker
  - Check when the executing code finished
  - Extract data from the SpiNNaker machine

The intention of the SpiNNTools software stack is to support a range of suitable applications executing on the SpiNNaker hardware by providing a flexible abstraction layer where the end user represents their problem as a graph which is then executed on the SpiNNaker machine without requiring such a low-level knowledge of how the machine works, thus overcoming the issues mentioned above. This concept is briefly mentioned as “The Uploader” in Brown et al. (2015), although, as will be demonstrated, the framework described herein is more complete in that it also:

- allows the user to express the generation of the data structures to be loaded (and possibly reloaded when changes have been made)
- controls the execution flow of the application where required
- aids in the storage and retrieval of data recorded during the execution
- extracts and presents provenance data which can be used to determine the correctness of the results.

## 4. SpiNNaker Core Software

This section discusses the software that runs on each core of the machine, together with the programming model. The ARM-968 cores can execute instructions from their code memory (ITCM) using the ARM or THUMB instruction sets; generally this code is generated from compiled C code using either the GNU *gcc* compiler^3^ or the ARM *armcc* compiler^4^. Although each SpiNNaker core is too simple and has too little local memory to be able to run an operating system such as embedded Linux, a library called *SARK* (Spinnaker Application Runtime Kernel) was designed with functions to interrogate, configure and control low level chip resources (Brown et al., 2015). It remains the closest thing that SpiNNaker has to an operating system. Additionally, an executable called *SCAMP* (Spinnaker Control And Monitor Program) is loaded to one of the cores on each SpiNNaker chip during the boot phase, allowing it to operate as a *monitor processor* through which the chip can be controlled. Supported functions include: the loading of compiled applications onto the other cores of the chip; the reading and writing of the SDRAM; and the loading of the SpiNNaker routing tables. SCAMP will also read the blacklist of its board and use this information to map out parts of the machine that are known to be faulty. During the phase in which the users application is compiled on a host workstation, the host software stack will talk to each monitor processor via SCAMP to obtain information on the state of its chip. The blacklist itself can be updated dynamically if components (cores, memory, router, or inter-chip links) are found to be faulty in future.

After SCAMP has been loaded onto one core on every chip of the machine, these cores then communicate with each other to work out the shortest pathway to every other chip, working around any known faults. They also establish where their nearest Ethernet port is located, so that a link to the host machine can be initiated. This communication makes use of the SpiNNaker Datagram Protocol (SDP) (Furber et al., 2014) which is encapsulated into UDP packets for communication with external machines. Communication out of the machine from any core is achieved by using *IP Tags*. The SCAMP monitor processor on each Ethernet chip maintains a list of up to 8 IP Tags which map between values in the tag field of the SDP packets and an external IP address and port. When a packet is received that is destined to go out via the Ethernet (identified in the SDP packet header), the tag table is consulted and a UDP packet is formed containing the SDP packet, using the IP address and port given in the table. The table can also contain Reverse IP Tags, where a UDP packet received from an external source is mapped from the UDP port in the packet to a specific chip and core on the machine, where the data of the packet is extracted and put in to an SDP packet before being forwarded to the specified core.

In addition to SARK, another library called *Spin1API* has been developed, providing support for event-driven applications (including our spiking neural network framework). Notionally, Spin1API sits on top of SARK in the software stack on each chip, further abstracting the details of the low level hardware from the application (Furber et al., 2014). Under this programming model an application running on a core is expected to remain dormant until awakened by an *event* that is implemented as an interrupt. The application is required to register callback functions during the start-up phase of the program, each associated with a given event type or source. When a registered event occurs the associated function is called and then the processor returns to sleep (or processes another event).

Examples of these events types include:

- Receiving packets of different types
- The expiration of an (optionally periodic) timer
- The completion of a memory transfer (DMA) between SDRAM and DTCM

The problem of writing code to run on the cores of the SpiNNaker machine is discussed in more detail in Brown et al. (2015), along with the types of applications which might be suitable to execute on the platform; in particular section 6.6 of this work gives some examples of applications which include neural simulation, electrical circuit simulation, finite difference solving, molecular dynamics, and large matrix mathematics. In the rest of this work, it is assumed that the application has already been designed to run in parallel and that SpiNNaker executables have been created for the application. The SpiNNTools software then works to map that parallel application on to the machine, execute it, and extract any results, along with any relevant data about the machine. In addition, the software also includes some additional C code libraries which run on top of the Spin1API to provide interfaces for some of the more advanced features supported. Note that it may be possible to automatically generate some of the SpiNNaker application code from higher level descriptions, as is described in Blundell et al. (2018), however it is expected that even in that case some of the base code will have been handwritten in advance, with only a template being filled in by the automatic code generation system.

## 5. Data Structures

In earlier sections we have examined the software running on the machine and summarized earlier versions of the main tool chain. We now turn to the software stack running on the host workstation which is written in Python. In this section, we describe the principle data classes used to represent the SpiNNaker machine and the user graph which are the principles inputs to the mapping process.

### 5.1. SpiNNaker Machines

**Figure 3:**
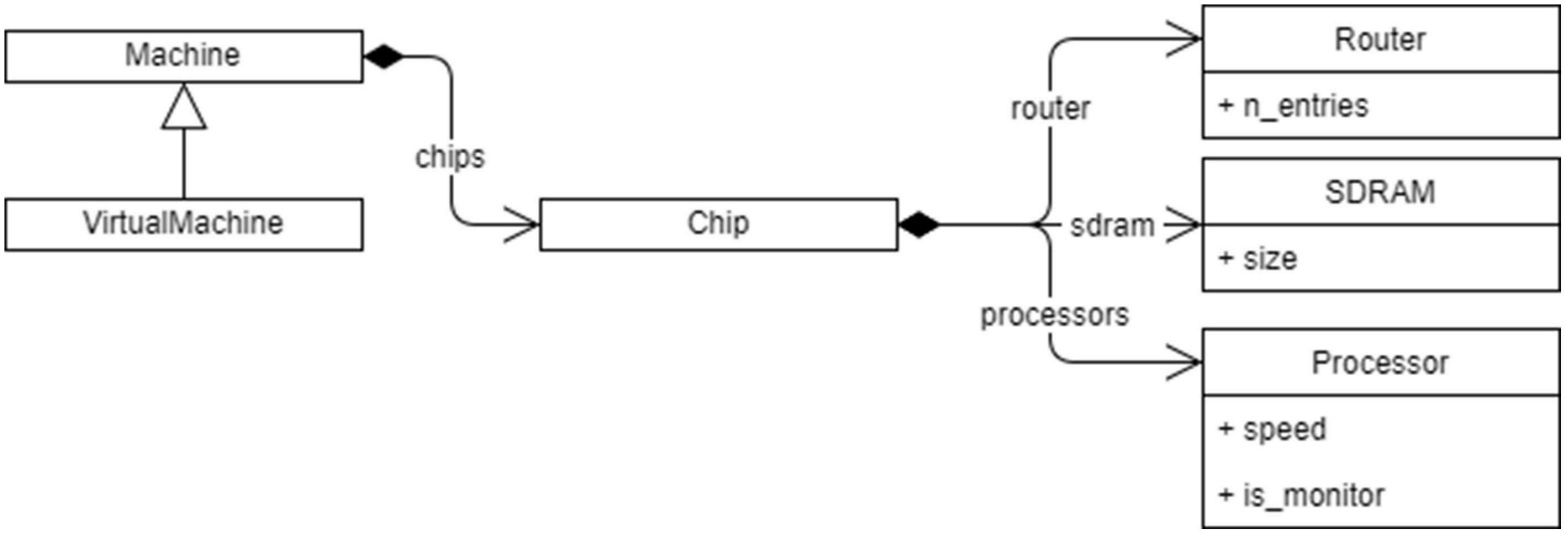
The Python class hierarchy for SpiNNaker Machine representation. The machine contains a list of chips, and each Chip contains a Router, SDRAM, and a list of Processor objects, each with their respective properties. A VirtualMachine can also be made that contains the same objects but can be identified as being virtual by the rest of the tools.

A SpiNNaker machine is represented as a set of Python classes as shown in Figure 3. An instance of the main Machine class contains instances of classes for each of the important details of the machine for mapping purposes, including:

- the chips, cores, and links available in the machine
- the clock speed of each core in MHz
- the SDRAM available on each chip in Bytes
- the number of routing entries available on each chip (in case some entries are used by the system software such as SCAMP)

As well as internally representing a physical, real-world machine with all its faults mapped out, the machine representation also allows the instantiation of a **virtual machine** for testing in the absence of connected hardware. The virtual machine can be further modified to simulate hardware faults and analyse the behavior of the software.

The connection of **external devices**, such as robotic devices like a silicon retina or a motor, to the machine is represented using *virtual chips*. A virtual chip will be given coordinates of a chip that doesn't exist in the physical machine and is marked as virtual. However, these coordinates are merely a means of giving the device a unique label—they are not used to map paths to the device directly since the device is only accessible through a single real chip and all attempts to access a “chip” marked as virtual will always go first to the real chip to which it is connected, then use the appropriate inter-chip link to connect to the external device.

### 5.2. Graphs

The graph in SpiNNTools allows the user to define units of computation and the communication between them. This is the extent to which the tools help with describing the parallel nature of the execution of the program; the tools do not help the user to solve issues of said parallelization, such as working out the dependencies in the computation beyond the communication.

**Figure 4:**
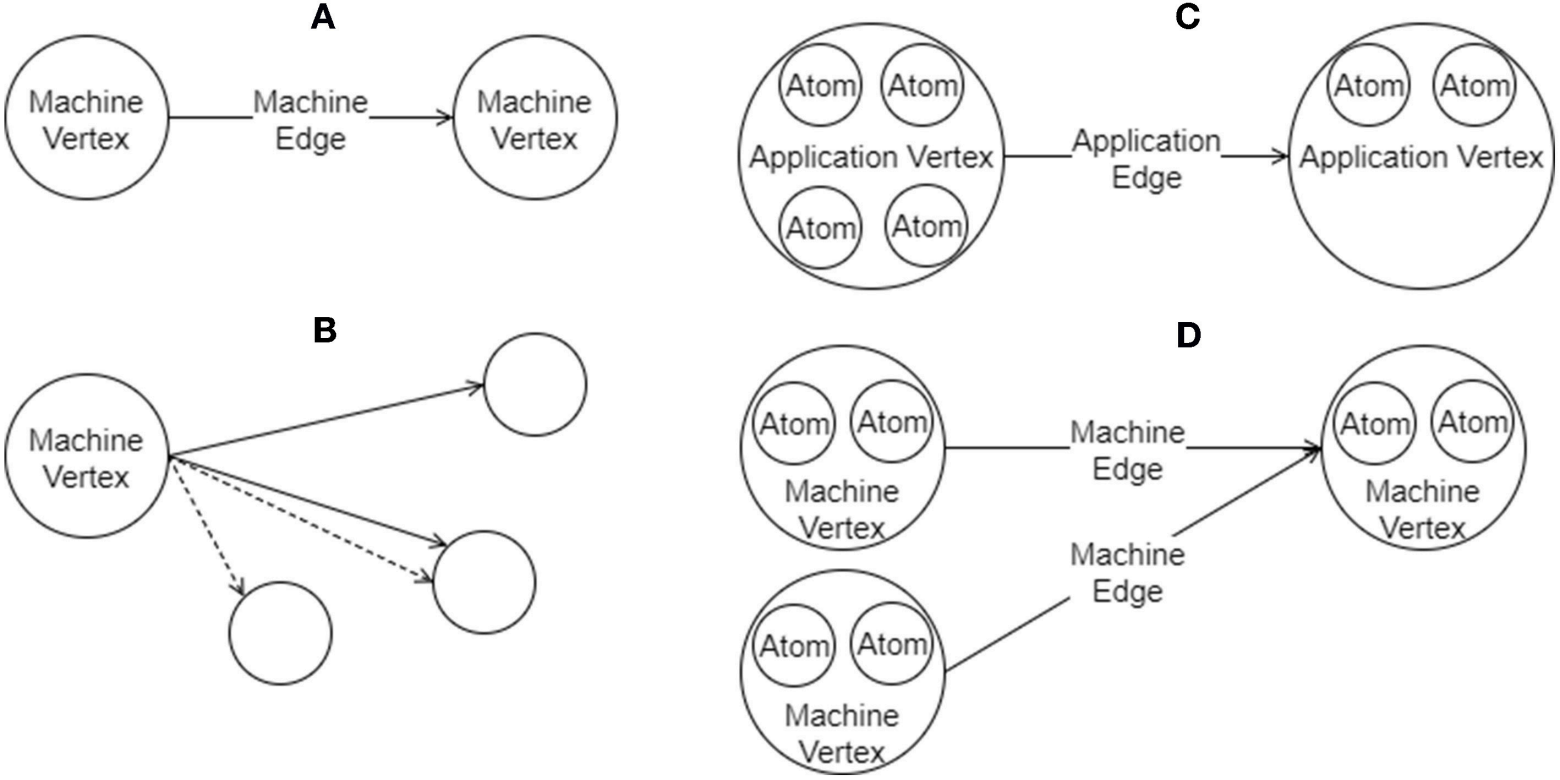
Graphs in SpiNNTools. **(A)** A Machine Graph made up of two Machine Vertices connected by a Machine Edge, indicating a flow of data from the first to the second. **(B)** A Machine Vertex sends two different types of data to two subsets of destination vertices using two different Outgoing Edge Partitions, identified by solid and dashed lines, respectively. **(C)** An Applications Graph made up of two Application Vertices, each of which contain two and four atoms, respectively, connected by an Application Edge, indicating a flow of data from the first to the second. (D) A Machine Graph created from the Application Graph in **(C)** by splitting the first Application Vertex into two Machine Vertices, each of which contain two atoms. The second Application Vertex has not been split. Machine Edges have been added so that the flow of data between the vertices in still correct.

A graph in SpiNNTools consists of vertices and directed edges between the vertices. The vertex is considered to be a place where computation takes place and as such each vertex has a SpiNNaker executable binary associated with it. An edge represents a communication pathway from a source, or *pre-vertex* to a target, or *post-vertex*. An additional concept is that of an *outgoing edge partition*, representing a group (in general a subset) of edges that all start at the same pre-vertex, partitioned according to some criteria such as message type. An example of outgoing edge partitions is shown in Figure 4B. The concept is useful to represent a multicast communication. Note that not all edges that have the same pre-vertex have to be in the same outgoing edge partition; there can be more than one outgoing edge partition for each source vertex, representing different message types which might be multicast to different sets of target vertices. Thus each outgoing edge partition has an identifier which can be used to identify the type of message to be multicast using that partition.

**Figure 5:**
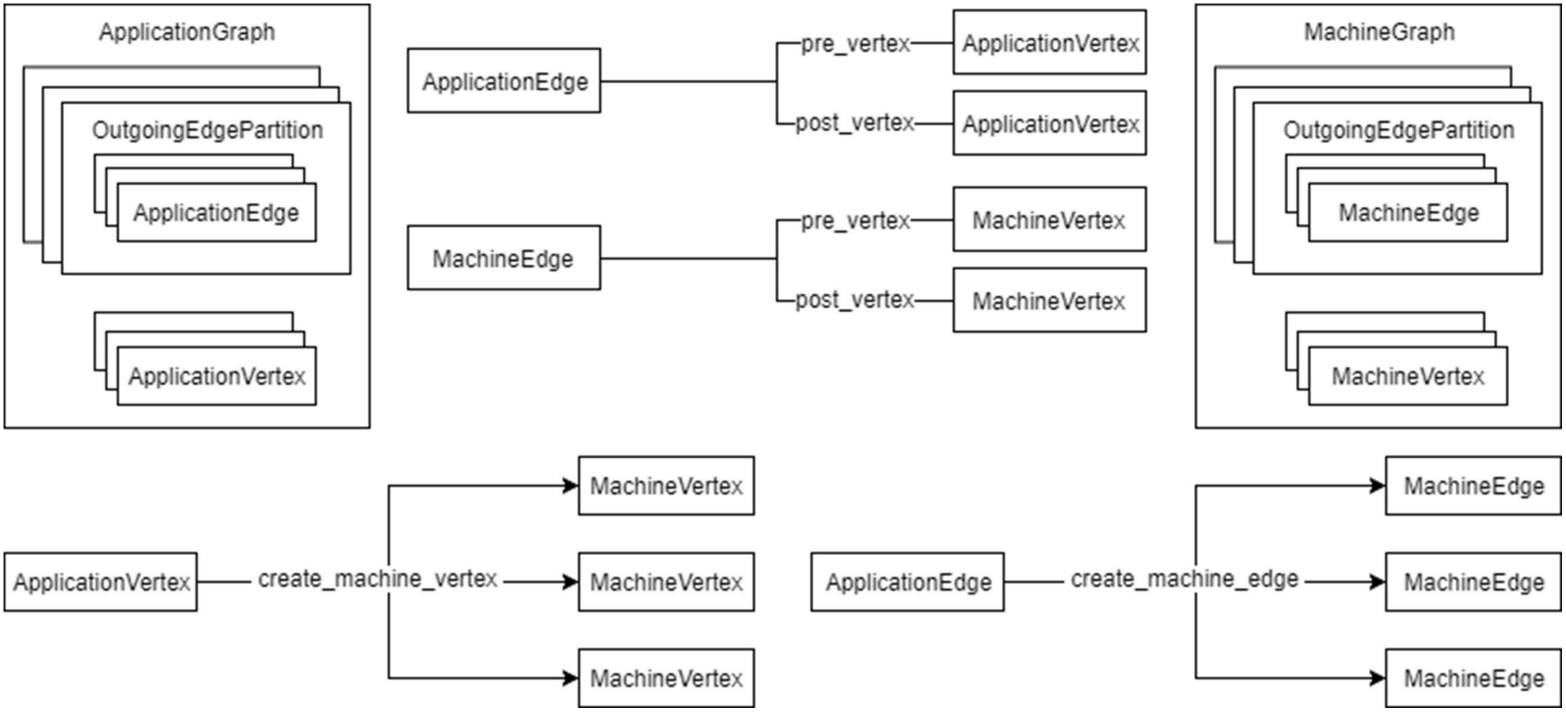
The relationship between the graph objects. An ApplicationGraph contains ApplicationVertex objects and OutgoingEdgePartition objects, which contain ApplicationEdge objects in turn. A MachineGraph similarly contains MachineVertex objects and OutgoingEdgePartition objects, which contain MachineEdge objects in turn. ApplicationEdge objects have pre- and post-vertex properties which are ApplicationVertex objects. Similarly MachineEdge objects and pre- and post-vertex properties which are MachineVertex objects. An ApplicationVertex can create a number of MachineVertex objects for a subset of the atoms contained therein and an ApplicationEdge can create a number of MachineEdge objects for a subset of atoms in the pre- and post-vertices.

There are two types of graph represented as Python classes in the tools (a diagram can be seen in Figure 5). Figure 4A shows an example of a *Machine Graph*, in which each vertex (known as a *Machine Vertex*) is guaranteed to be able to execute on a single SpiNNaker processor. A *Machine Edge* then represents communication between cores. In contrast, Figure 4C shows an example of an *Application Graph*, in which each vertex (known as an *Application Vertex*) contains one or more atoms, each of which represents the minimum unit of computation into which the application can be split. Of course, it may be possible to run multiple atoms of an Application Vertex on a single core. Each edge (known as an *Application Edge*) represents communication of data between the groups of computational atoms; if one or more of the atoms in an Application Vertex communicates with one or more atoms in another Application Vertex, there must be an Application Edge between those Application Vertices. It is not guaranteed that all the atoms on an Application Vertex fit on a single core, so the instruction code for Application Vertices should know how to process a subset of the atoms, and how to handle a received message and direct it to the appropriate atom or atoms. The graph classes support adding and discovering vertices, edges and outgoing edge partitions.

As the vertices represent the application code that will run on a core they have methods to communicate their resource requirements in terms of the amount of DTCM and SDRAM required by the application, the number of CPU cycles used by the instructions of the application code in order to maintain any time constraints, and any IP Tags or Reverse IP Tags required by the application. The Application Vertex provides a method that returns the resources required by a continuous range or slice of the atoms in the vertex; the value returned is specific to the exact range of atoms requested, allowing different atoms of the vertex to require different resources. The Application Vertex additionally defines the maximum number of atoms that the application code can execute on each core of the machine (which might be unlimited), and the total number of atoms that the vertex represents. These measurements allow the Application Vertex to be broken down into one or more Machine Vertices as seen in Figure 4D. The Application Vertex class has a method for creating Machine Vertex objects for a continuous range of atoms. A Machine Vertex can return the resources it requires in their entirety.

The graphs additionally support the concept of a **Virtual Vertex**, a vertex that represents a device connected to a SpiNNaker machine. The Virtual Vertex indicates which chip the device is physically connected to, allowing the tool chain to work with this information to include the device in the network. As with the other vertices, there is a version of the Virtual Vertex for each of the machine and application graphs.

## 6. The SpiNNTools Tool Chain

**Figure 6:**
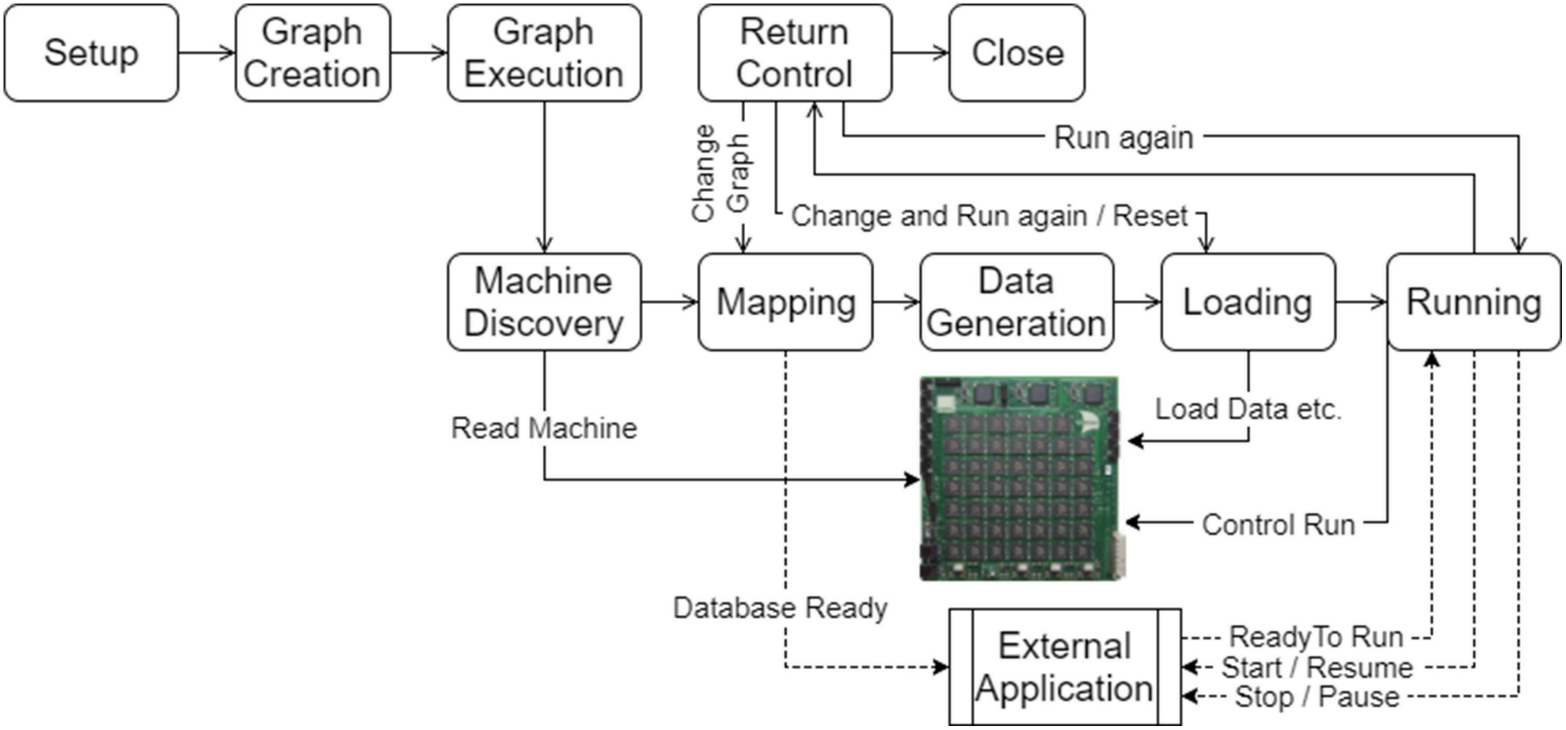
The execution work flow of the tools on the host machine in use within an application. Following the setup of the tools, the graph is created and then executed. The execution results in machine discovery, mapping of the graph on to the machine, generation of data for the application on the machine, loading of the data and application binaries and then running of the application. Once the run is complete, control is returned to the application. The flow can then be resumed at different stages depending on what has changed since the last execution. An external application can also interact with the tools and be sent messages that allow it to keep in synchronization with a simulation running on the machine.

The aim of the tool chain is to control the execution of a program described as a graph on the SpiNNaker machine. The tool chain software is executed on the host machine in several steps as shown in Figure 6, and detailed below. The software has been designed to run on any host machine that can run Python and can install the appropriate libraries which includes machines that run Windows, Linux, and Mac OS X. The host machine must also be able to communicate with the Ethernet connection(s) of the SpiNNaker machine.

### 6.1. Setup

The first step in using the tools is to initialize them. As part of this process, the user can specify appropriate configuration parameters such as the time step of the simulation and the location where binary files can be located on the host machine. The tools then set up the initially empty graphs and read in configuration files for further options such as the SpiNNaker machine to be used. Options are separated out in this way to make a clear distinction between script-level parameters that might apply no matter where the script is run (such as the time step of the simulation) and user-level parameters that will be different per-user but likely to be common across multiple scripts for that user (for example the SpiNNaker machine to be used).

### 6.2. Graph Creation

The graph creation step is part of the user's execution script, in which vertices and edges to either an application or machine graph may be added. It is an error to add vertices or edges to both of these structures. The tool chain keeps track of the graph as it is built up.

Users can extend the vertex and edge classes to add additional information relevant to their own application. Typically, users will need to extend the vertex classes (which are provided as abstract classes) to ensure that the implementation provides the resources required as well as the name and **execution type** of the SpiNNaker-compiled binary file; the execution type indicates to the tools whether the binary was compiled with additional library support to work with additional features of the tool chain (see later). Other additional features of the tool chain can also be used by extending additional abstract classes. These will be detailed in the later sections.

### 6.3. Graph Execution

Graph execution takes the created graph and performs the step required to execute it on the machine. Methods are provided to run for a specified period of time, to run until a completion state is detected (such as all cores being in an exit state having completed some unit of work), or to run *forever* meaning that execution can be stopped through a separate call to the tools at some indeterminate time in the future, or the execution can be left on the machine to be stopped outside of the tools by resetting the machine. The graph execution itself consists of several phases shown in the lower half of Figure 6 and detailed below.

#### 6.3.1. Machine Discovery

The machine discovery algorithm is shown in Algorithm 1. Further details are discussed below.

**Algorithm 1:**
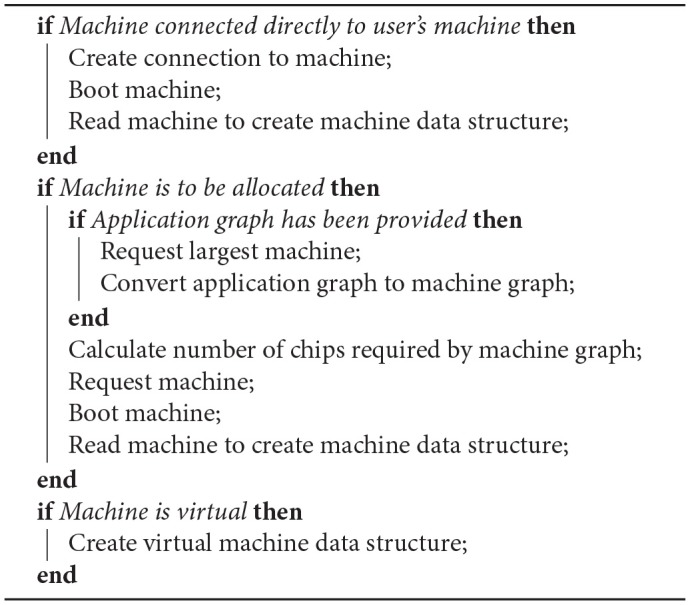
Machine Discovery algorithm

If the user has configured the tools to run on a single physical machine then that machine is contacted and booted (if necessary). Communications with the machine then take place to discover the chips, cores and links available. This data is used to build up a Python machine representation that can be interrogated by the rest of the tools.

If a machine is to be allocated, the tools must first work out how big a machine to request, by working out how many chips the user-specified graph requires. If a machine graph has been provided then the number of cores is exactly the number of vertices in the graph. The resources must still be queried as the SDRAM requirements of the vertices might mean that not all of the cores on each chip can be used. For example, a graph consisting of 10 machine vertices, each requiring 20 MB of SDRAM, and thus 200 MB of SDRAM overall, will not fit on a single chip in spite of their being enough cores.

If an application graph is provided, it must first be converted into a machine graph to determine the size of the machine required by executing some of the algorithms in the mapping phase (see below).

#### 6.3.2. Mapping

The mapping phase takes the graph and maps it on to the discovered machine. In other words the vertices of the graph are assigned to cores on the machine and edges of the graph are converted into communication paths though the machine. Additionally, other resources required by the vertices are mapped on to machine resources to be used within the simulation.

If the graph is an application graph it must first be converted to a machine graph. The conversion may have been done during the machine discovery phase as described previously. To allow the prior conversion, the algorithm(s) used in the *graph partitioning* process are kept separate from the rest of the mapping algorithms.

Once a machine graph is available it is mapped to the machine through a series of phases that generate data structures to be used later in the process, including:

- A set of **placements** detailing which vertex is to be run on which core of the machine
- A set of **routing tables** detailing how communications over edges are to pass between the chips of the machine
- A set of **routing keys** detailing the key or range of keys that must be sent by each vertex in order to communicate over each outgoing edge partition starting at that vertex
- A set of **IP tags and reverse IP tags** to identify which external communications are to take place and through which Ethernet-connected chip

Note that once a machine has been discovered, mapping can be performed entirely separately from the machine using the Python machine data structures created. It is interesting to note here that an alternative strategy would be to have the mapping process make use of the machine itself, turning the graph mapping problem into a highly parallel relaxation algorithm running on the cores of the SpiNNaker machine. This functionality is not implemented at present and we leave the design of such an algorithm to future work.

Mapping information can be stored in a database by the system to allow external applications which interact with the running simulation to decode any live data received. As shown in Figure 6, the applications can register to be notified when the database is ready for reading and to receive other notifications later in the execution cycle.

#### 6.3.3. Data Generation

The data generation phase creates a block of data to be loaded in to the SDRAM for each vertex which contains any parameters and other relevant data from the Python-described vertices to be used by the application code to be executed on the machine. The vertices can make use of the mapping information above as appropriate when generating the data. For example, the **routing keys** and **IP tags** allocated to the vertex can be passed to ensure that the correct keys and tags are used in transmission. The graph itself could also be used to determine which **routing keys** are to be received by the vertex and so set up appropriate actions to take upon receipt of these keys.

A user with a vertex that generates data should extend the AbstractGeneratesDataSpecification abstract class, requiring the user to implement the generate_data_specification method. Some support for data generation is provided by the tools at both the Python level (where data can be generated in *regions*) and at the C code level (where library functions are provided to access these regions). A class that extends the aforementioned abstract class will be provided with an object that they can use to write their specification. Callable methods on this object include:

- reserve_memory_region, allowing the user to create regions to which data can be written
- switch_write_focus, allowing the user to switch attention between different regions when writing new data
- write_data, allowing the user to append a single value to the currently selected region and to specify the required data type (data format conversion is then performed automatically); for example, having supplied a 32-bit floating point number, the user can choose to write a signed or unsigned 32-bit, 16-bit or 8-bit integer, or a signed or unsigned fixed point number
- write_array, allowing the user to append an array of values to the currently selected region.

There is currently no support in the tools to maintain synchrony between the data required by the vertices (in the C code running on the SpiNNaker machine) and the data created by the data generation phase (in the host-based Python code). Users must therefore take care to generate the data in the correct order and using the types appropriate for their data. It is also important to note that the tools do not currently provide support for padding in data structures—users should be aware of the padding requirements in C data structures when writing sequences of values of different bit-lengths. Support for ensuring a match between the Python data generation and the C code is to be developed in the future.

#### 6.3.4. Loading

The loading phase prepares the physical machine for execution by loading the **routing tables** generated on to each chip of the machine, the application data into the SDRAM of the machine, the **IP tags and reverse IP tags** into the Ethernet chips and the application code (executable) into the ITCM of the cores. The application loader can send executable code to multiple cores in the machine in a single operation, significantly speeding up this part of the loading process.

#### 6.3.5. Running

The running phase starts off the actual execution of the simulation on the SpiNNaker machine and, if necessary, monitors the execution until complete as shown in Algorithm 2. Before execution, the tools wait for the completion of the setup of any external applications that have registered to read the mapping database. The external applications are then notified when the application is about to start and to finish, allowing them to keep in synchronization with the simulation.

**Algorithm 2:**
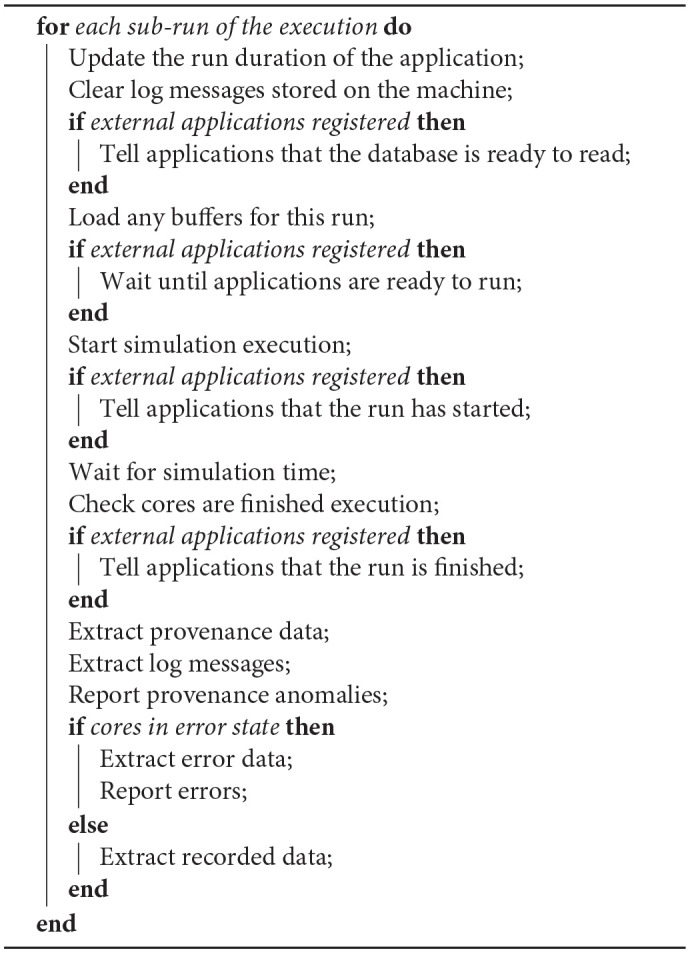
Algorithm for running for a fixed time.

Once a run is complete, application recorded data and provenance data is extracted from the machine. The provenance data includes:

- Router statistics, including dropped multicast packets.
- Core-level execution statistics, including information on whether the core has kept up with timing requirements.
- Custom core-level statistics. The information contained depends on the application, but might include such things as the number of spikes sent in a neural simulation, or the number of times a certain condition has occurred.

During provenance extraction, each vertex analyses the data and reports any anomalies. The log files from each core can also optionally be extracted and can then be analyzed, printing any *error* or *warning* lines.

If a run is detected to have failed in any way, the tools will attempt to extract information about that failure. One possible failure mode that a core may have entered an error state at some point during processing. It is also possible for a core to fail to enter the completion state after the expected simulation time (with some margin), perhaps indicating a problem during execution. Log files will be automatically extracted in such cases and analyzed as previously discussed. Any cores that are still alive will also be asked to stop and extract any provenance data so that this can also be analyzed in an attempt to diagnose the cause of the error.

**Figure 7:**
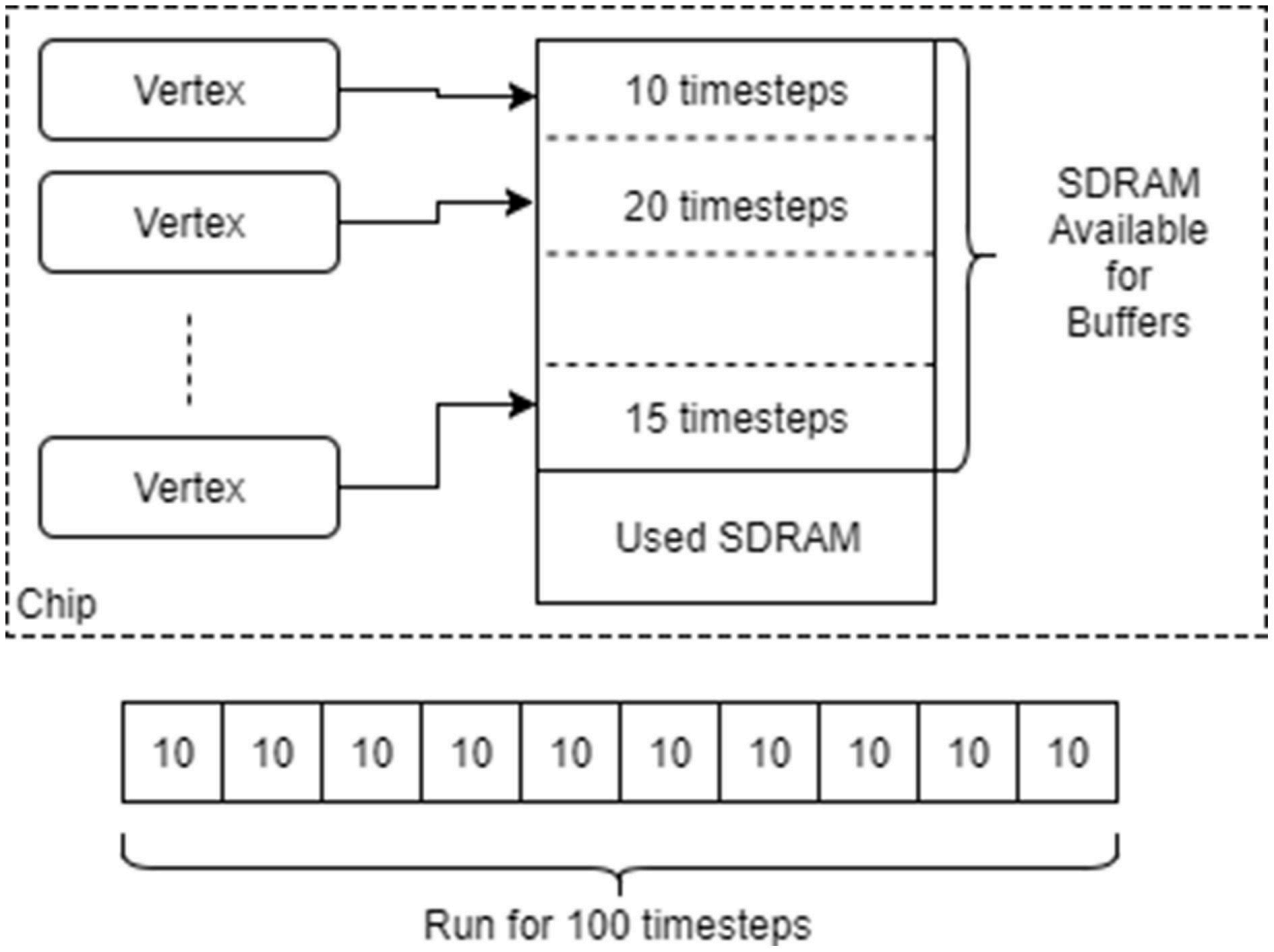
Running vertices with recorded data. The SDRAM space remaining on each chip after it has been allocated for other things is divided up between the vertices on that chip. Each is then asked to provide an upper bound on the number of time steps for which it can be run before filling up its local SDRAM. The minimum of these upper bounds is taken as the maximum length of each simulation interval. A single run is split into many such intervals, with simulation pausing after each one to extracted and then delete the recorded data.

The run may be split into several sub-runs to allow for the limited SDRAM on the machine, as shown in Figure 7. After each run cycle, any recorded data is extracted from the SDRAM and stored on the host machine, after which the recording space is flushed and the run cycle restarted. Additional support within the binary of the vertex is required to allow a message to be sent to the core to increase the run duration and to reset the recording state. These operations are provided in the form of C code library functions, with callbacks to allow the user to perform additional tasks before resuming execution. Additionally, the tools can be set up to extract and clear the core logs after each run cycle to ensure that the logs do not overflow.

The length of each run cycle can be determined automatically by the tools by working out the SDRAM available on each chip after data generation has taken place, dividing the space equally between the vertices on the chip and then asking how many units of time each vertex can run for given that space. The minimum such time is taken and used as the unit for each cycle with any left over time then used up in the last run cycle. To ensure that there is some space for recording, vertices can also reserve a minimum recording space. This functionality requires that the vertex implement an additional interface to provide the required information to allow the tools to make these decisions.

### 6.4. Return of Control / Extraction of Results

Once the run cycles have completed the tools return control to the executing script, after which the user can interact with the graph again. At this time, interactions might include extracting any recorded data (see later), or making changes to the graph and/or the parameters before resuming the simulation. The effect of any change is detailed below.

### 6.5. Resuming / Running Again

The user can choose to resume the execution of the simulation or to reset the simulation and start it again. At this point, the tools must decide which of the aforementioned steps need to be run again, using the algorithm described in Algorithm 3.

**Algorithm 3:**
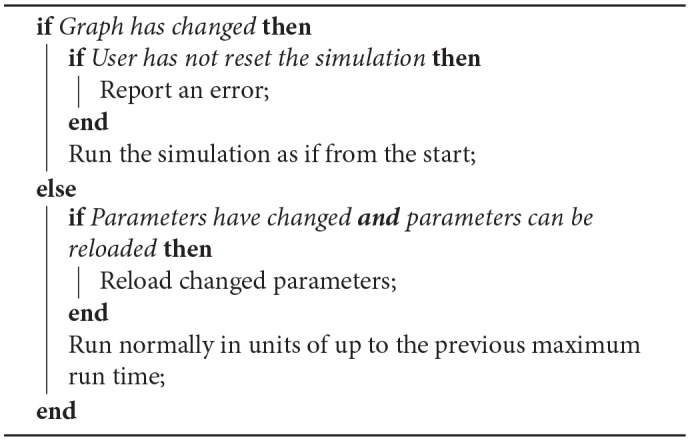
Algorithm for resuming simulation.

Any change to the graph, such as the addition of a vertex or edge, is likely to require that the mapping phase take place again which may even result in a new machine being required should the size of the graph increase to this degree. Such a change will mean that all the other phases will also have to be executed again.

If the parameters of any of the vertices or edges have been changed, the vertex can be set up to allow the reloading of these changes where the change won't increase the size of the data, such as a change in neuron state update parameters in a neural network. Any increase in the size of the data, such as an increase in the number of synapses in a neural network, would likely require a remapping of the graph on to the machine since the SDRAM is likely to be packed in such a way as to prohibit the expansion of the data for a single core; it is left to the vertex to make this decision however.

The case where no changes have been made to the graph or the parameters can be considered an extension of the aforementioned ability for the tools to run the code in phases. The minimum time calculated previously is respected again here and the tools will then run in cycles of that unit of time. Note that if the first run-time is shorter than that required to fill the remaining SDRAM space (and thus only one run cycle was required previously) the buffers will have already been initialized to record for only that amount of time. A future extension to this functionality is to allow the buffers to be sized to use up all of the remaining SDRAM regardless of the run time, and then allow runs in units of less than or equal to the time that uses all of the space.

### 6.6. Closing

Once the user has finished simulating and extracted any data, they can tell the tools that they are finished with the machine by closing the tools. At this point, the tools reset and release any machines that have been reserved. Any recorded data that has not be extracted by this point will no longer be available. If the tools were told to run the network for an indeterminate length, the extraction and evaluation of any provenance data would happen during this call, before the machine is released.

### 6.7. Algorithms and Execution

In order to run each of the above phases, the tools execute a series of algorithms. The algorithms consume various inputs that are made available by the tools and by other algorithms and produce various outputs. These inputs and outputs are not constrained in any other way; thus algorithms are not constrained to produce only one output. For example, this could be useful in *mapping* where an algorithm could be made to produce both placements and routing tables that have been optimized together. This approach is more flexible and more powerful than a more restrictive methodology, in which each algorithm can only perform one of a pre-determined list of tasks and produce only a single output type, such as having a specific algorithm for generating placement and another for generating routing tables.

**Figure 8:**
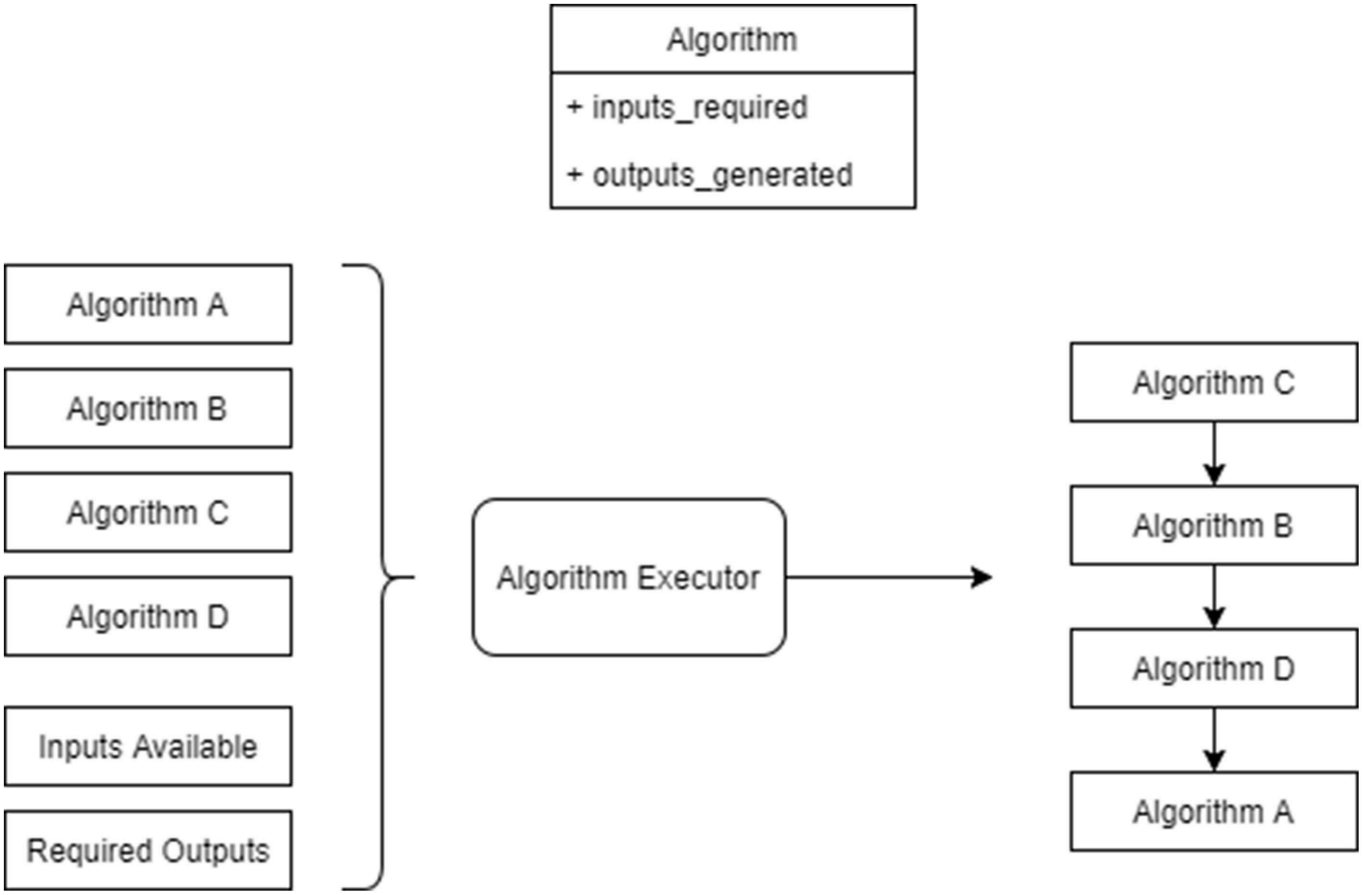
Algorithms being run by the algorithm execution engine. The executor is provided with a list of algorithms to run, a set of input items and a set of output items to produce. It then produces a workflow for the algorithms accounting for their inputs required and outputs produced.

To support this form of execution, the tools implement a workflow execution system, shown in Figure 8 that examines the algorithms to be run in terms of the inputs required and outputs generated in order to compute an execution order for the algorithms. Input and output *tokens* are also supported. These indicate implicit inputs and outputs, for example one token might be generated by an algorithm to represent that data has been loaded on to the machine and another can require that the data loading has been completed before it executes.

The algorithms themselves are not discussed here in detail other than those mentioned above. A more detailed discussion of the mapping algorithms is discussed in Heathcote (2016). The tools also include algorithms for routing table compression, which is discussed in Mundy et al. (2016). Many of the other algorithms are currently simplistic in nature; these can be replaced in the future should other algorithms be found to perform more efficiently and / or effectively.

### 6.8. Data Recording and Extraction

**Figure 9:**
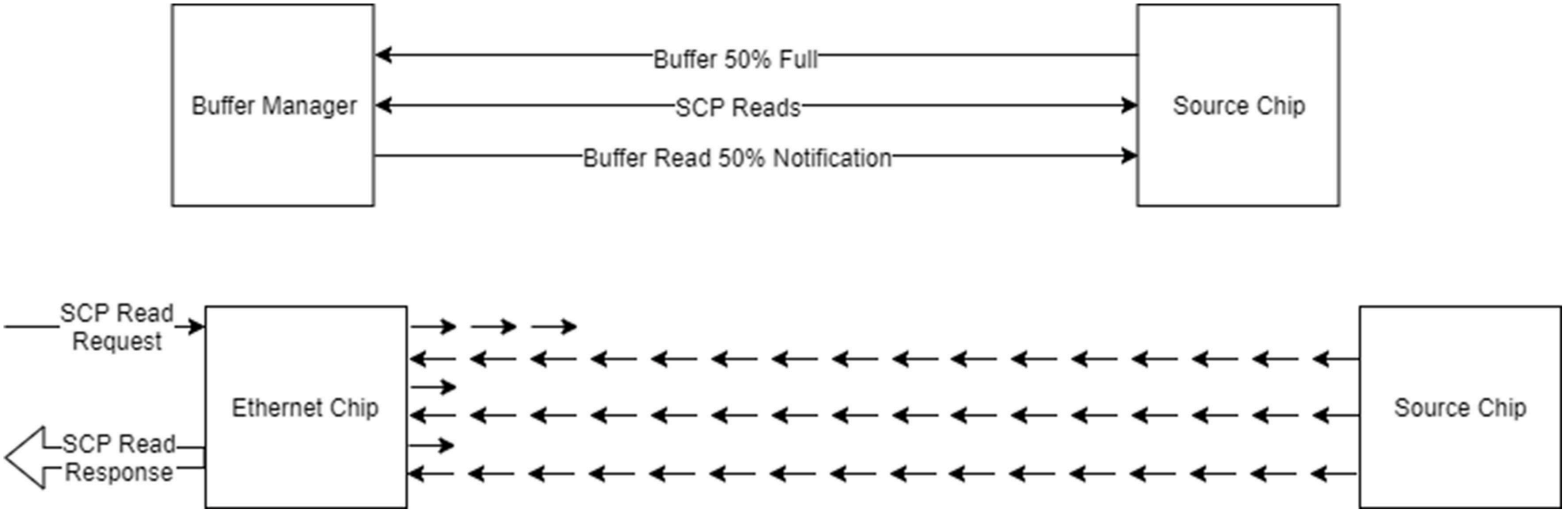
Data buffering and extraction. **Top:** The buffer manager is used to read back recorded data during execution; when the buffer contains some data, the buffer manager is notified and attempts to read the data, notifying the data source once this has been done to allow the space to be reused. **Bottom:** Data reading done using SCAMP; each read of up to 256 bytes is further broken down in to a number of request and read cycles on the machine itself, where the packets used contain only 24-bits of data each.

As mentioned previously, the tools support the recording of data in such a way as to cope with the limited nature of the SDRAM on the machine. A *buffer manager* is provided that is used to keep track of and store the buffers of data as they are extracted from the machine and to support the live extraction of buffers whilst the simulation is running, as shown in Figure 9 (Top). Cores configured with the provided library can contact the host machine when the recording space is getting full and the tools can then attempt to extract the data. In general the bandwidth of the Ethernet of the machine is not fast enough for this to be effective, resulting in data loss and so in practice pausing the simulation whilst the buffers are extracted works better.

The SCAMP software supports the reading of SDRAM through SDP messages using a request and response system, where each SDP message can request the reading of up to 256 bytes of data. Additionally, to transmit the SDP message to chips which are not connected to the Ethernet, each message must be broken down in to SpiNNaker network messages, and then reconstructed on receipt; an overview of how this process works is shown in Figure 9 (Bottom). The speed of reading data using this method is around 8 Mb/s when reading from the Ethernet chip and around 2 Mb/s when reading from other chips. The speed can be improved upon by having the host send a number of packets before waiting for responses, which has the effect of filling the time waiting for the response by sending packets and results in speeds of around 35 Mb/s when reading from the Ethernet chip and around 8 Mb/s when reading from other chips.

### 6.9. Live Interaction

We have previously mentioned that external applications can interact with a live simulation, making use of the mapping database. Additional support for this interaction is provided by the tools, split into live data output and live data input.

**Figure 10:**
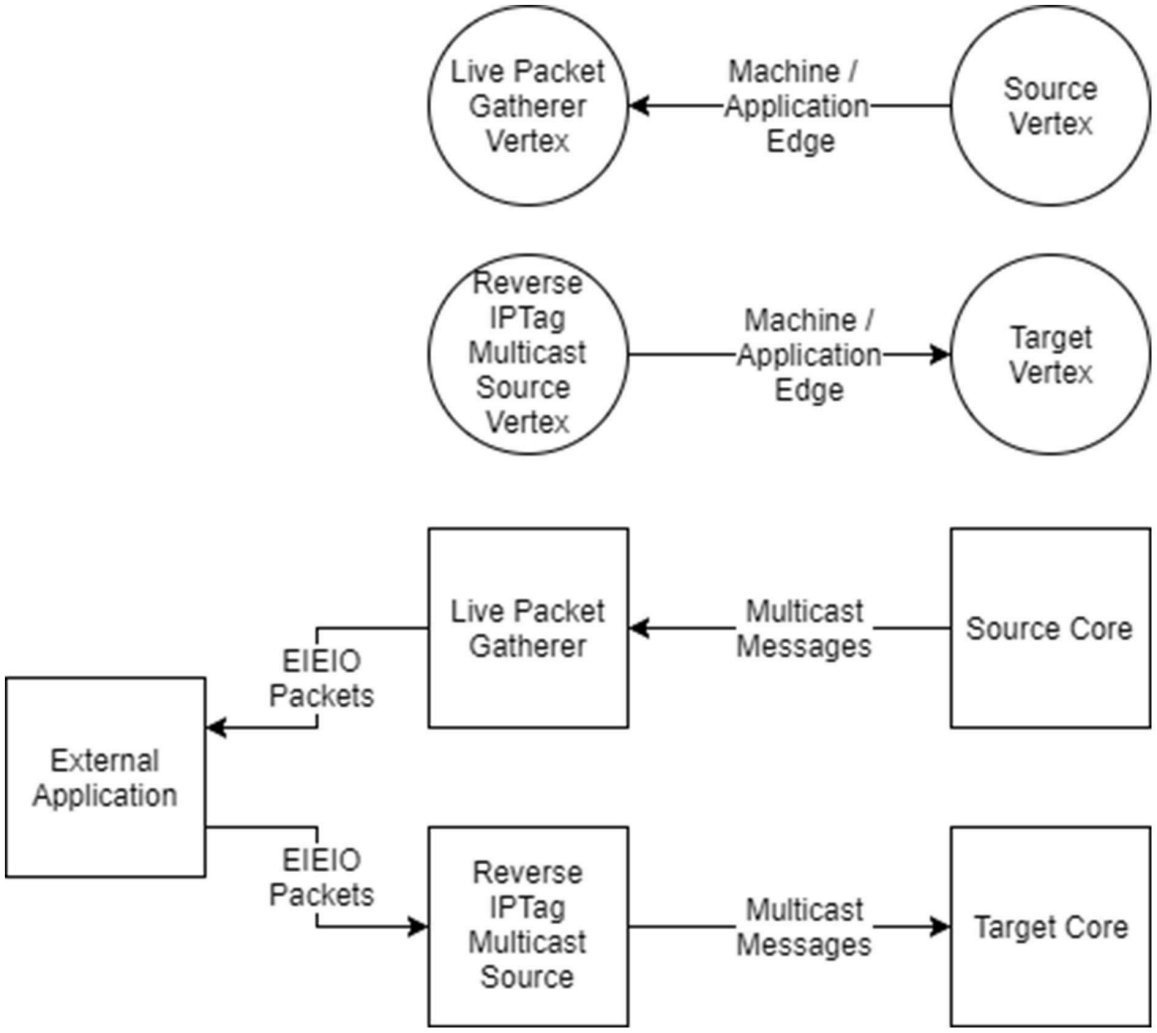
Live interaction with vertices. **(Top)** To indicate that live output is required, an edge is added from the vertex which is the source of the data to the Live Packet Gatherer vertex in the graph. To indicate the live input is required an edge is added from the Reverse IP Tag Multicast Source vertex to the target of the data in the graph. **(Bottom)** The effect of adding the edges to the graph is that multicast messages will be sent from the core (or cores) of the source vertex to the core running the Live Packet Gatherer, which will then wrap the messages in EIEIO packets and forward them to a listening external application. EIEIO packets received from an external application will be decoded by the Reverse IP Tag Multicast Source core and sent onward as multicast messages to the target core (or cores).

Live data output support is performed by a vertex called the *Live Packet Gatherer* which will package up any multicast packets it receives and send them as UDP packets using the EIEIO protocol (Rast et al., 2015). It is configured by adding edges to the graph from vertices from which output data is required which has the advantage of being able to tap into the existing multicast streams that are already being used to communicate within the machine, as shown in Figure 10.

Live data input support is provided via a vertex called the *Reverse IP Tag Multicast Source*, which will unpack and send multicast packets using the same EIEIO protocol. Edges can be added that direct traffic from this source to the vertices which are to receive the messages.

External applications that would like to make use of the live support can read the mapping database to determine the multicast keys to be received and decoded in the case of live output, or to be sent in the case of live input. Support for reading the database and receiving and sending keys is provided in the tools in both Python code and host-based C++ code.

### 6.10. Dropped Packet Re-injection

As mentioned in section 2, when a packet is dropped an interrupt is raised allowing a core to detect and capture the dropped packet. The tools include software that runs on the SpiNNaker machine to detect the interrupt and then capture the packets that have been dropped and store them until a time at which the router is no longer blocked, at which time they can be safely sent onwards. Re-injection of dropped packets helps in those applications where the reliable transmission of packets is critical to their operation.

There is only one register within the SpiNNaker hardware to hold a dropped packet. If a second packet is dropped it will be completely unrecoverable, but an additional flag is set so the re-injection core can detect this and count such occurrences to be reported the user at the end of the execution. Thus there is a record that something may not be correct in the simulation results.

## 7. Use Cases

In this section we will look at two example applications that can be described as a graph and that work well with the SpiNNaker machine. These applications will be used to show how the tools work to support the use of the machine.

### 7.1. Conway's Game of Life

**Figure 11:**
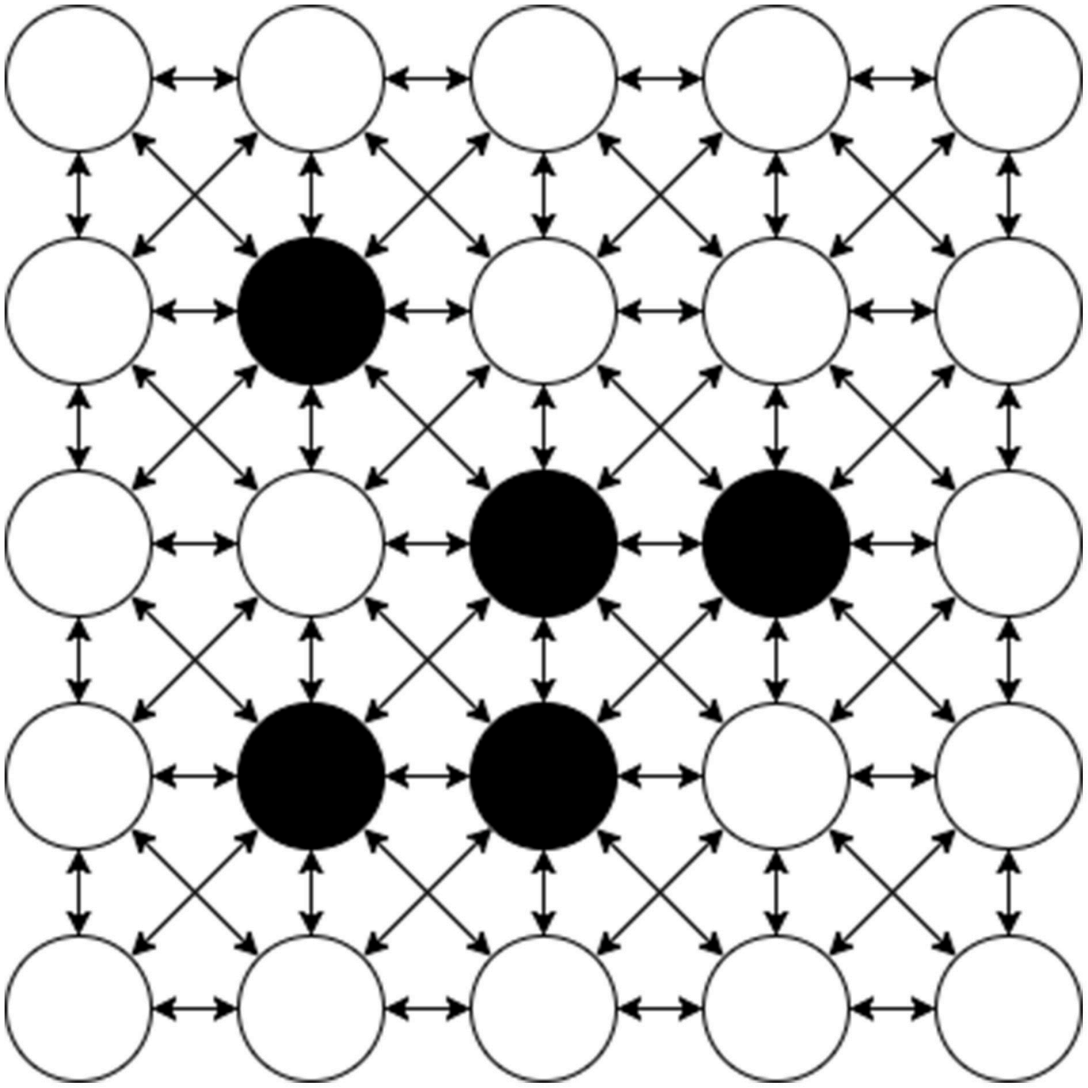
Conway's Game Of Life on a 5 × 5 grid as a Machine Graph. Every Machine Vertex is connected to each of it's 8 neighbors bi-directionally requiring two Machine Edges for each bi-directional connection. The initial state of each Vertex is either alive (black) or dead (white).

Conway's Game of Life (Gardner, 1970) consists of a collection of cells which are either alive or dead based on the state of their neighboring cells. A diagram of an example Machine Graph of this problem is shown in Figure 11. The vertices of the graph of this application are each a cell in the game; given the state of the surrounding cells this cell can compute whether it is dead or alive in each step, and then send that to its neighbors. It similarly receives the state of the neighbors as they are transmitted and uses this to update its own state. The edges of the graph are thus between adjacent cells in a grid, where each vertex is connected bidirectionally to its eight surrounding neighbors. The game proceeds in synchronous phases, where the state of cells in a given phase are all considered at the same time.

Graphs of this form are highly scalable on the SpiNNaker system since the computation to be performed at each node is fixed and the communication forms a regular pattern that does not increase as the size of the board grows. Therefore once working, it is likely that any size of game can be built up to the size of the available machine. Conway's Game of Life works well as an archetype of a class of problems, which includes finite element analysis (Bi, 2018) problems. Problems in this class should be implementable on the SpiNNaker machine provided that the data to be transmitted can be broken down into SpiNNaker packets and that the instructions required for the problem can fit within the instruction memory.

The application code of Conway's Game of Life updates the cell based on the state of the surrounding cells once per time step of the simulation based on the received state from the surrounding cells and then send its own new state out using the given key. It can also record its state at each time step in the simulation. The set up of this application is as follows:

- A Conway vertex is created which extends the machine vertex class
- A number of Conway vertices are added to the graph to make up the board. These are stored in such a way that finding an adjacent vertex in the grid is easy
- A machine edge is added between each pair of adjacent vertices, in each direction
- Each machine vertex generates data for the vertex which includes the key to be sent by that vertex and the number of time steps to run for
- Each machine vertex can tell the tools how many time steps it can run for given an amount of SDRAM available for recording
- Each machine vertex contains code to read the state that is recorded at each time step using the Buffer Manager

The code for an implementation of Conway's Game of Life on SpiNNaker is available as part of the SpiNNakerGraphFrontEnd release^5^ and consists of:

- conways_cell.c, the SpiNNaker C code which executes on the cores of the machine updating the state as the simulation progresses
- Makefile, a file to be processed by GNU Make to build the C code
- conways_basic_cell.py, the Python code describing the Conway's Machine Vertex. This includes the following methods:
  - get_binary_file_name which returns the name of the SpiNNaker binary generated from the c code
  - generate_machine_data_specification which generates the data required by the C code, where information such as the SpiNNaker routing key to transmit data with is extracted
  - get_data which extracts the recorded state information from the machine
  - resources_required which returns mainly the SDRAM required by the vertex for holding the parameters, including that required for recording
  - get_minimum_buffer_sdram_usage which returns the minimum space to reserve for recording to ensure that some recording can be done
  - get_n_timesteps_in_buffer_space which indicates how many time steps can be recorded in a given space of SDRAM, to allow the tools to determine the length of a run cycle
- conways_partitioned.py, a script that executes a specific example of Conway's Game of Life on a 7 by 7 grid which calls the tools setup, builds a graph of several instances of the Conways Machine Vertex connected together using Machine Edge instances as described above. The run method is then called to execute the graph, followed by the extraction and display of the results (which are a series of ASCII grids of the game board as it progresses)

**Figure 12:**
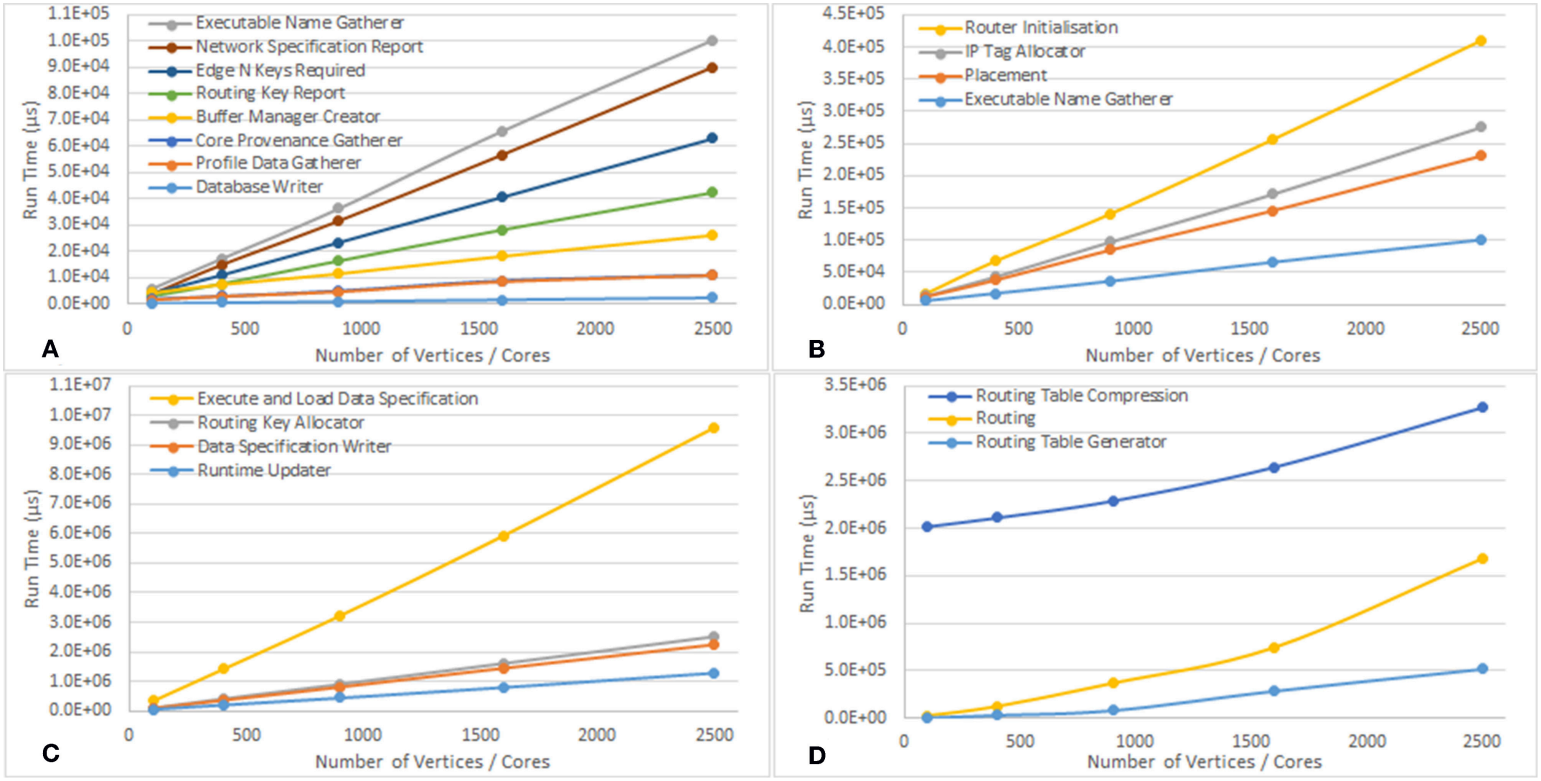
Graphs showing how the run time of the various parts of the tool chain scale with the number of vertices in the Conway's Game of Life simulation. **(A–C)** Show the algorithms which scale linearly, and have been separated to account for the different scales of the run times. **(D)** Shows how some of the algorithms scale non-linearly.

An example output from the execution of the script is included in the Supplementary Material. Table 1 shows the run time in μs of the various stages of execution of the script once the run is started until the run is complete. Some observations from this data include the following:

- The Machine Allocator takes a non-linear amount of time in relation to the problem size because of the way in which the machine allocation server works when interacting with multiple clients. An additional 5 s are added by the service between the allocated machine being powered on and the machine being returned to the tools to ensure that all boards in any allocated machine are fully powered on
- The Machine Generator also takes a non-linear time in relation to the problem size because the machine also has to be booted and this can require a number of retries depending on network conditions
- The Router Provenance Gatherer execution time is proportional to the number of boards in the machine because it reads the data from the routers of every chip in the machine, not just the ones that were used in the simulation, to ensure that nothing unexpected happened on the other routers such as stray packets being received
- The Tag Loader scales with the number of boards in the machine because tags are only loaded on to the Ethernet-connected chip of each board
- The Virtual Chip Allocator and Database Notification algorithms take around the same time in all cases because they have no work to do in this run (there are no external communications)
- The time for Load Executable Images does not vary with the size of the problem as expected since executable code for each binary only needs to be copied to the machine once after which it can be rapidly replicated on to the required cores
- The Application Runner takes the same time across all runs because simulation is run for the same duration
- The Routing, Routing Table Compression, and Routing Table Generation algorithms appear to have a more complex non-linear scaling, shown in Figure 12D. These algorithms may therefore require refinement as problems of larger scale are encountered to avoid them dominating the overall execution time
- The remaining algorithms scale linerly with the number of vertices in the simulation as shown in Figures 12A–C

**Table 1:** Average run times over 10 runs of the SpiNNTools algorithms, in order of execution, when executing Conway's Game of Life on various grid sizes, broken down into phases of execution.

**CONWAY'S GAME OF LIFE SIZE**					
Number vertices / cores	100	400	900	1,600	2,500
Number of edges	800	3,200	7,200	12,800	20,000
Number of chips used	7	28	57	103	160
Number of boards used	1	1	3	3	6
**MACHINE DISCOVERY (μs)**					
Machine allocator	8,711,589	10,799,373	10,175,818	12,076,720	6,731,204
Machine generator	8,040,498	8,470,635	6,963,204	5,674,133	10,230,590
Virtual chip allocator	86	85	85	93	91
**MAPPING (μs)**					
Network specification report	3,705	14,953	31,667	56,875	90,130
Placement	12,078	37,719	84,610	145,805	230,735
Routing	29,002	128,009	370,468	747,004	1,682,054
IP tag allocator	12,715	42,762	96,990	171,202	275,011
IP tag report	945	553	624	590	581
Edge N keys required	4,164	11,085	23,426	40,706	62,953
Routing key allocator	100,638	410,597	906,178	1,608,309	2,515,832
Routing key report	3,007	7,767	16,441	28,254	42,442
Routing table generator	5,533	30,833	81,163	285,620	517,796
Executable type locator	1,602	3,451	6,390	10,444	15,819
Buffer manager creator	4,280	7,453	11,580	18,214	26,184
**DATA SPECIFICATION (μs)**					
Data specification writer	91,863	361,230	810,060	1,447,989	2,247,400
Graph provenance gatherer	3,073	8,069	16,864	30,550	45,736
**LOADING (μs)**					
Router initialization	16,681	67,168	141,138	256,470	410,147
Executable name gatherer	5,787	17,242	36,305	65,802	100,398
Routing table compression	2,017,828	2,110,796	2,287,198	2,644,092	3,270,905
IP tag loader	3,617	3,352	8,953	9,757	18,219
Load data	335,054	1,411,766	3,221,989	5,937,052	9,586,231
Load executable images	962,930	969,326	965,471	979,613	1,014,863
**RUNNING (μs)**					
Runtime updater	57,257	199,705	441,409	798,570	1,281,730
Database writer	243	563	1,007	1,672	2,442
Database notification	1,311	1,161	1,287	1,400	1,213
Application runner	303,362	303,475	303,622	303,897	304,273
Core provenance gatherer	1,964	3,012	5,045	8,908	11,060
Router provenance gatherer	76,984	75,238	243,184	240,695	530,201
Profile data gatherer	1,681	2,955	4,763	8,535	10,945

*Note that the data recorded starts when the user calls the run function, and stops at the end of the Running phase; in particular, setup, graph creation and data extraction times are not included here as they are not currently recorded by the tools. The number of chips and boards used by the simulations is also reported to show the scaling. These numbers were obtained on a server with 128GB RAM, an Intel Xeon E5520 2.27GHz CPU and an SSD drive, running in the same machine room as the SpiNNaker machine, connected to it via a 1GB/s interface*.

Once the graph is built, the script starts the execution of the graph during which the tools will obtain a machine description to be used with the machine graph to work out a placement of each of the vertices and a routing of the edges between these placements, along with an allocated key for each of the vertices. The tools will then ask each vertex how many time steps it can record for, based on the available SDRAM after placement is complete and the resources used on each chip can therefore be determined. Each vertex will then be asked to generate its data, based on the mapping and timing information. The tools will then load the generated data on to the machine along with the routing tables and application code and start the execution of the cores and then wait an appropriate amount of time for the cores to stop, before finally checking their status. Assuming the cores complete the simulation successfully, control will return to the script which can then request and display the recorded states from each of the vertices in an appropriate way.

A future version could have a Conway vertex that can have multiple cells within each machine vertex which would then be an application vertex of cells. The whole problem can then be expressed using a single large application vertex which would represent the whole game board and an application edge for each of the 8 directions of connectivity, each in its own Outgoing Edge Partition to indicate that different keys are required for each of the directions. The application code of the vertex would now have to cope with the reception of multiple neighbor states which would make the application code itself more complex; for example, it would have to cope with multiple incoming keys from each direction, each of which would target a different cell within the grid. A list of target cells stored for each key received would be sufficient. The application vertex implementation would also have to now be able to work out the resources required by a range of atoms in each machine vertex which could include some basic data that all machine vertices use as well as some resources specific to each atom.

Another possible extension to this application is to extract the state during execution to be displayed as the application progresses. The Live Packet Gatherer vertex (described above) would need to be added to the graph, along with an edge from each of the Conway vertices to this vertex. The script would then indicate before executing the graph that there is an external application that would like to receive the data. The receiving application would be notified when the mapping database had been written, at which point it can set up a mapping between multicast keys received and positions in the game board, responding when it has completed its own setup. The tools would then notify the application that the simulation is starting and the application will then receive the same state messages as the vertices receive. This information can be used to update the display of the game board.

### 7.2. Spiking Neural Networks

The SpiNNaker machine is primarily designed to simulate spiking neural networks (Furber et al., 2013) and indeed the tools are included within the version 4.0.0 release of the sPyNNaker software (Rowley et al., 2017b). A more detailed description of the framework for simulating neural networks is found in Rhodes et al. (2018), but we examine here the application from the perspective of the tools. SpiNNTools has been part of the sPyNNaker software since conception and this release is successfully deployed as part of the EU Flagship Human Brain Project Collaboratory^6^, and has been used successfully in a number of simulations in previous works (e.g., Senk et al., 2017; Albada et al., 2018; Rast et al., 2018; Sen-bhattacharya et al., 2018).

**Figure 13:**
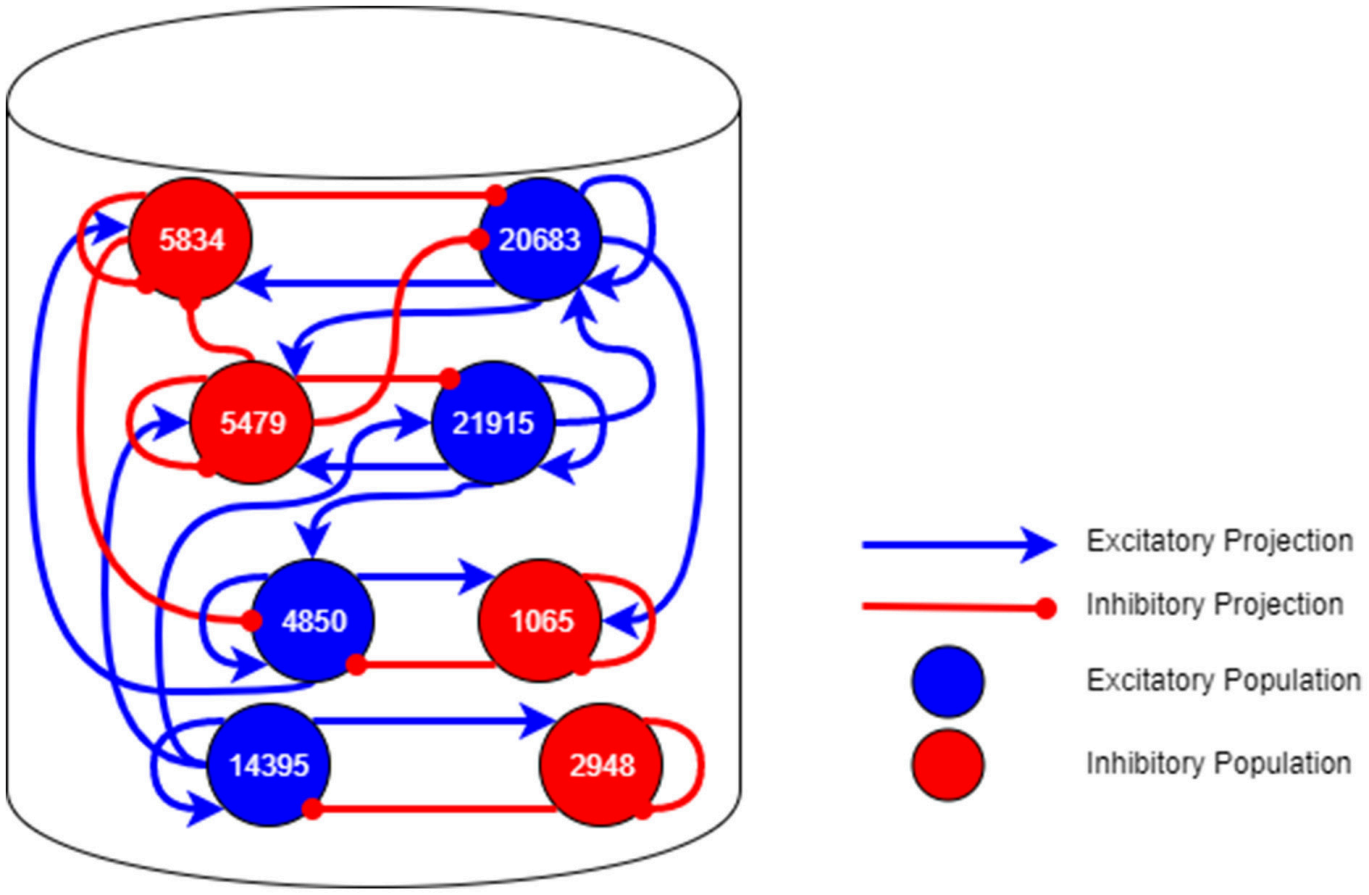
A neural network topology of a 1*mm*^2^ area of cortical micro-column found within the mammalian brain.

As an example, we consider the simulation of a cortical micro-column found within mammalian brains, details of which can be found in Albada et al. (2018). The model consists of the neurons within a structure underneath a 1*mm*^2^ area of the surface of the generic early sensory cortex (Potjans and Diesmann, 2014). Figure 13 shows the groups of neurons (Populations) in this network and connectivity between them (Projections). In a spiking neural network, the vertices are groups of point neurons (as a single core can simulate more than one neuron); the computation required is the update of the neuron state in response to spikes received from connected neurons. The edges are then groups of synapses between the neurons over which spikes are transmitted. These are potentially unidirectional and are likely to be more heterogeneous in nature than the regular grid pattern seen in Conway's Game of Life.

The computation required to simulate each neuron at each time step in the simulation is generally fixed. The remaining time is then dedicated to processing the spikes received, the number of which depends on the how many neurons are sending spikes to the core and the activity of those connected neurons. This isn't known in advance in general and so some flexibility in the system with respect to the amount of computation available at each node is necessary to allow the application to work in different circumstances. Once the spike rate is known for a given network, the system could potentially be reconfigured with additional cores allowing that network to be simulated in less time overall. The platform is designed to be able to simulate 1,000 neurons on each core each with 1,000 incoming synapses spread across up to 10,000 source neurons spiking at up to 10 Hz each; the framework doesn't enforce these requirements, and the software is not yet optimized to handle this case in general, so users should keep this in mind when designing neural networks to execute with sPyNNaker. To demonstrate this, the results found in Albada et al. (2018) show that the current sPyNNaker implementation is not able to simulate the cortical column example in real-time due to the specifics of this network being outside of the design specifications detailed above. The tools helped to make it possible to simulate the network at a slower speed however, giving a starting point to improving on the implementation in the future.

We now look at how sPyNNaker makes use of SpiNNTools. The sPyNNaker software includes SpiNNaker application code that can process a number of neurons on each core. The application will be set up with a series of synaptic matrices, one for each core that it can receive from, which the application can use to demultiplex the messages received from other cores, and direct them to the appropriate receiving neuron. A more detailed description of the application code, including the mechanism for demultiplexing the incoming data, is found in Rhodes et al. (2018).

In addition to the neuron application code, a Poisson spike generator code has also been created, which can generate spikes randomly with a given rate using a Poisson process (Gerstein and Mandelbrot, 1964). This is only intended to generate spikes rather than to receive them, though it might generate spikes for a number of source neurons. All vertices can record the spikes generated by each of the neurons, irrespective of whether they were generated by a Poisson process or through or normal neuron dynamics.

The setup for this application is as follows:

- An application vertex is created for each group of simulated neurons (known as a Population), and another for each of the Poisson generators
- A matching machine vertex is created for each of the above application vertices. These machine vertices will be generated during the graph conversion and each will be expected to take as a parameter the range of atoms assigned to it
- An application edge is created to describe the connectivity between groups of neurons; the source vertex will be either a Poisson vertex or a neuron vertex but the target can only be another neuron vertex as described above. The edge will contain details of the neuron-to-neuron connectivity to allow the generation of the synaptic matrices
- The neural network is described as a set of neuron application vertices and Poisson source application vertices with neural application edges between them
- Each machine vertex can generate data for the vertex it represents, including the parameters of the group of neurons, and, in the case of the neuron vertex, the source keys for each of the neurons it is to receive data from
- Each machine vertex can tell the tools how many time steps it can run for given a space in SDRAM. Note that the spike recording regions are sized assuming that every neuron spikes on every time step to ensure that the buffers don't overfill should this actually happen
- Each machine vertex contains code to read spikes from the recorded data using the Buffer Manager

Once the graph describing the neural network has been built the script will start execution of the graph, which will in turn result in the execution of the simulation of the network. Firstly the application graph is converted into a machine graph by asking the application vertices how much resource they require for different ranges of neurons. As with the Conway example, the tools will then go through the various stages until the execution of the simulation is complete. Control will then return to the script and the user will be able to extract any recorded spikes and process these.

The live output example described in the Conway's use case also works similarly well in the neural networks use case. Another extension more relevant here is the connection of an external device to the machine which will then be controlled by the network (e.g., a robotic device), as was the subject of Rast et al. (2018). In this case an extension of the virtual application vertex is made to represent the device and added to the application graph. Now, when the graph is executed the tools will add a virtual chip to the discovered machine and a placement constraint to the vertex, placing the device on the virtual chip. The tools will then operate as normal with edges to and from the device being routed as appropriate. The algorithms will now recognize that the chip is virtual and will make use of the adjacent chip when necessary. Loading will recognize that the chip is virtual also, and so it will not attempt to load any data onto it; this is further enforced with the virtual vertex *not* implementing the interface for the writing of data.

**Figure 14:**
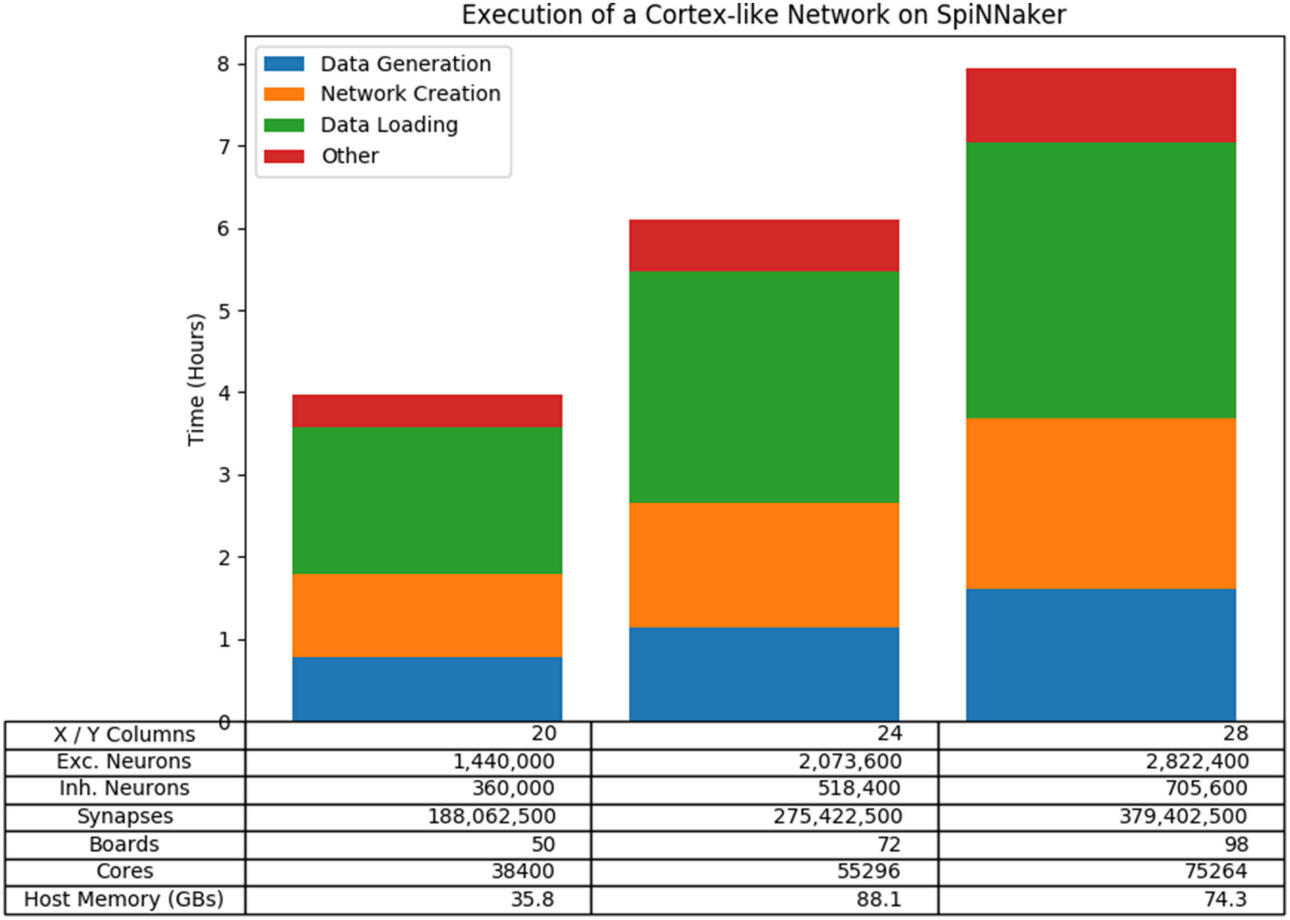
Results of running a scalable cortical-like network at various scales on the machine. The overall run time is shown, broken down to show the longest phases of the run which are the Data Generation phase and the Data Loading phase. Also included is the time taken to create the network; that is the time taken between setting up the tools until the graph begins to execute.

To test the performance of the neural network use case in more detail, we constructed a scalable cortex-like neural network that fits within the original design specifications of the machine^7^. The network consists of a number of *regions* connected together, where each region consists of a 2-dimensional grid of columns and each column consists of a number of layers of pairs of inhibitory and excitatory populations of neurons, similar to the cortical micro-column. The script allows the variation of a number of parameters, but for simplicity we scaled the number of columns within each region using a square arrangement, with the number of layers and the number of regions fixed at 6. Figure 14 shows the results of three of these tests which run on 50, 72, and 98 boards, respectively. The results show that the tools are capable of running on large SpiNNaker machines and that the overall execution time (including the building of the graph) scales linearly with the network size in this case.

## 8. Future Work

Although the SpiNNTools software provides many useful features there are still many improvements that can be done. Some of those include:

- An application graph can only include application vertices. Some utility vertices, such as the Live Packet Gatherer and the Reverse IP Tag Multicast Source may be more suited to being described as machine vertices. To avoid having to provide both an application and machine vertex for those utilities, it might be better to allow an application graph to contain machine vertices which are then simply copied to the machine graph during the conversion.
- It would be useful to provide some support at the C code level for demultiplexing messages from multiple atoms. This would make writing code that supports application vertices easier.
- Data structures are currently manually written from the Python data generation and then read back from the C code. This process could be streamlined by having a structure object in Python that can be used both to generate a C header file including the structure and to write the data and to manage the SDRAM usage of the data in the Python code.
- The speed at which data is transferred to and from the machine using SDP packets is quite slow, particularly when speaking to chips that are not directly connected to the Ethernet. Mechanisms whereby data is sent to the Ethernet chip using multicast may be able to improve this. Data transfer could be further improved by having cores on the Ethernet chip use the Ethernet adaptor directly rather than having to go through SCAMP.

## 9. Conclusions

We have described a software system—**SpiNNTools**—that can be used to help to execute a problem described as a graph on SpiNNaker Neuromorphic machines. We have described how the tools operate at the high level by proceeding through a series of steps that result in the mapping of the graph onto the machine, the execution of that graph and the obtaining of results from the execution. We have then shown how this applies to two example problems and the work that is required to make each work on the machine. We have shown that although SpiNNTools does not solve all the problems it abstracts away many of the steps required in the operation of the SpiNNaker machine, therefore making that operation easier. Applications that make use of the tools will also see the benefits of any improvements without requiring large updates to their code-base.

## Author Contributions

This work presents the latest version of the SpiNNTools software package produced by the SpiNNaker group at the University of Manchester, UK. The current SpiNNaker software team is comprised of CB, DF, AG, OR, and AS, and led by AR, all of whom have made significant contributions to SpiNNTools. SD, DL, and LP are researchers within the SpiNNaker group, and worked on earlier versions of SpiNNaker software, and provided assistance with low-level programming and hardware interactions during performance analysis. AR led the research and wrote the manuscript, while SF leads the SpiNNaker project and supervised this work. All authors reviewed and refined the final manuscript.

## Conflict of Interest Statement

The authors declare that the research was conducted in the absence of any commercial or financial relationships that could be construed as a potential conflict of interest. The reviewer AP declared a past co-authorship with several of the authors AR, DL, AS to the handling editor.

## Footnotes

^1^The software is open source and can be downloaded from https://github.com/SpiNNakerManchester

^2^Available from https://github.com/SpiNNakerManchester/spinnaker_tools/releases

^3^https://developer.arm.com/open-source/gnu-toolchain/gnu-rm/downloads

^4^https://developer.arm.com/products/software-development-tools/compilers/legacy-compiler-releases

^5^The full set of files of the example described here can be found at https://github.com/SpiNNakerManchester/SpiNNakerGraphFrontEnd/tree/4.0.0/spinnaker_graph_front_end/examples/Conways/partitioned_example_b_no_vis_buffer

^6^https://collab.humanbrainproject.eu

^7^The script for this network can be found at https://github.com/SpiNNakerManchester/SpiNNakerPerformance/blob/pynn8/performance_testing/performance_tests/big_cortical_network_8.py
