# Supplementary material for: SpiNNTools: The Execution Engine for the SpiNNaker Platform
Source: Front Neurosci. 2019 Mar 26;13:231. doi: 10.3389/fnins.2019.00231 (PMC6444189; doi:10.3389/fnins.2019.00231)
Supplement: Supplementary file 1 [file Data_Sheet_1.PDF]

```
['/localhome/zzalsar4/SpiNNTools4.0.0/local/lib/python2.7/site-packages/spinn_front_end_common/interface/spinnaker.cfg', '/localhome/zzalsar4/SpiNNTools4.0.0/local/lib/python2.7/site-packages/spinnaker_graph_front_end/spiNNakerGraphFrontEnd.cfg', '/localhome/zzalsar4/.spiNNakerGraphFrontEnd.cfg']
2019-01-29 14:35:39 INFO: Read config files: /localhome/zzalsar4/SpiNNTools4.0.0/local/lib/python2.7/site-packages/spinn_front_end_common/interface/spinnaker.cfg, /localhome/zzalsar4/SpiNNTools4.0.0/local/lib/python2.7/site-packages/spinnaker_graph_front_end/spiNNakerGraphFrontEnd.cfg, /localhome/zzalsar4/.spiNNakerGraphFrontEnd.cfg
2019-01-29 14:35:39 INFO: Will search these locations for binaries: /localhome/zzalsar4/SpiNNTools4.0.0/local/lib/python2.7/site-packages/spinn_front_end_common/common_model_binaries : /localhome/zzalsar4/SpiNNTools4.0.0/lib/python2.7/site-packages/spinnaker_graph_front_end/examples/Conways/partitioned_example_b_no_vis_buffer
2019-01-29 14:35:39 INFO: Setting time scale factor to 1.
2019-01-29 14:35:39 INFO: Setting machine time step to 1000 micro-seconds.
Created spalloc job 1593152
2019-01-29 14:35:39 INFO: Created spalloc job 1593152
Waiting for board power commands to complete.
2019-01-29 14:35:39 INFO: Waiting for board power commands to complete.
2019-01-29 14:35:48 INFO: Time 0:00:08.939157 taken by SpallocAllocator
2019-01-29 14:35:48 INFO: Creating transceiver for 10.2.225.1
2019-01-29 14:35:48 INFO: Working out if machine is booted
2019-01-29 14:35:50 INFO: Attempting to boot machine
2019-01-29 14:35:52 INFO: Found board with version [Version: SC&MP 3.1.0 at SpiNNaker:0:0:0 (built Fri Jan 27 10:23:34 2017)]
2019-01-29 14:35:52 INFO: Machine communication successful
2019-01-29 14:35:52 INFO: Detected a machine on ip address 10.2.225.1 which has 836 cores and 116 links
2019-01-29 14:35:52 INFO: Time 0:00:04.311940 taken by MachineGenerator
2019-01-29 14:35:52 INFO: Time 0:00:00.000087 taken by MallocBasedChipIDAllocator
2019-01-29 14:35:52 INFO: Starting execution process
2019-01-29 14:35:52 INFO: Time 0:00:00.003671 taken by NetworkSpecificationReport
Placing graph vertices
|0%                          50%                          100%|
 ============================================================
2019-01-29 14:35:52 INFO: Time 0:00:00.014793 taken by RadialPlacer
Routing
|0%                          50%                          100%|
 ============================================================
2019-01-29 14:35:52 INFO: Time 0:00:00.029834 taken by RigRoute
Allocating tags
|0%                          50%                          100%|
 ============================================================
2019-01-29 14:35:52 INFO: Time 0:00:00.014401 taken by BasicTagAllocator
Reporting Tags
|0%                          50%                          100%|
 ============================================================
2019-01-29 14:35:52 INFO: Time 0:00:00.002411 taken by TagReport
Getting number of keys required by each edge using machine graph
|0%                          50%                          100%|
 ============================================================
2019-01-29 14:35:52 INFO: Time 0:00:00.003682 taken by EdgeToNKeysMapper
Allocating routing keys
|0%                          50%                          100%|
 ============================================================
2019-01-29 14:35:52 INFO: Time 0:00:00.097475 taken by MallocBasedRoutingInfoAllocator
Generating Routing info report
|0%                          50%                          100%|
 ============================================================
2019-01-29 14:35:52 INFO: Time 0:00:00.002737 taken by routingInfoReports
Generating routing tables
```

```
|0%                          50%                         100%|
 ============================================================
2019-01-29 14:35:52 INFO: Time 0:00:00.005211 taken by BasicRoutingTableGenerator
Finding executable_start_types
|0%                          50%                         100%|
 ============================================================
2019-01-29 14:35:52 INFO: Time 0:00:00.001451 taken by LocateExecutableStartType
Initialising buffers
|0%                          50%                         100%|
 2019-01-29 14:35:52 INFO: Listening for packets using tag 1 on 0.0.0.0:34866
============================================================
2019-01-29 14:35:52 INFO: Time 0:00:00.004226 taken by BufferManagerCreator
Generating data specifications
|0%                          50%                         100%|
 ============================================================
2019-01-29 14:35:53 INFO: Time 0:00:00.103132 taken by GraphDataSpecificationWriter
Getting provenance data from machine graph
|0%                          50%                         100%|
 ============================================================
2019-01-29 14:35:53 INFO: Time 0:00:00.003049 taken by GraphProvenanceGatherer
Preparing Routing Tables
|0%                          50%                         100%|
 ============================================================
2019-01-29 14:35:53 INFO: Time 0:00:00.018408 taken by RoutingSetup
Finding binaries
|0%                          50%                         100%|
 ============================================================
2019-01-29 14:35:53 INFO: Time 0:00:00.008920 taken by GraphBinaryGatherer
Running routing table compression on chip
|0%                          50%                         100%|
 ============================================================
2019-01-29 14:35:55 INFO: Time 0:00:02.013446 taken by MundyOnChipRouterCompression
Clearing tags
|0%                          50%                         100%|
 ============================================================
Loading Tags
|0%                          50%                         100%|
 ============================================================
2019-01-29 14:35:55 INFO: Time 0:00:00.007236 taken by TagsLoader
Executing data specifications and loading data
|0%                          50%                         100%|
 ============================================================
2019-01-29 14:35:55 INFO: Time 0:00:00.362981 taken by HostExecuteDataSpecification
Loading executables onto the machine
|0%                          50%                         100%|
 ============================================================
2019-01-29 14:35:56 INFO: Time 0:00:00.830290 taken by LoadExecutableImages
2019-01-29 14:35:56 INFO: Running for 1 steps for a total of 50 ms
2019-01-29 14:35:56 INFO: Run 1 of 1
Updating run time
|0%                          50%                         100%|
 ============================================================
2019-01-29 14:35:56 INFO: Time 0:00:00.063007 taken by ChipRuntimeUpdater
2019-01-29 14:35:56 INFO: Time 0:00:00.000228 taken by DatabaseInterface
2019-01-29 14:35:56 INFO: Time 0:00:00.001631 taken by NotificationProtocol
2019-01-29 14:35:56 INFO: *** Running simulation... ***
Loading buffers (0 bytes)
|0%                          50%                         100%|
 ============================================================
2019-01-29 14:35:56 INFO: *** Awaiting for a response from an external source to state its
ready for the simulation to start ***
2019-01-29 14:35:56 INFO: *** Sending start / resume message to external sources to state
```

```
the simulation has started or resumed. ***
2019-01-29 14:35:56 INFO: *** Awaiting for a response from an external source to state its ready for the simulation to start ***
2019-01-29 14:35:56 INFO: Application started - waiting 0.15 seconds for it to stop
2019-01-29 14:35:56 INFO: *** Sending pause / stop message to external sources to state the simulation has been paused or stopped. ***
2019-01-29 14:35:56 INFO: Time 0:00:00.303282 taken by ApplicationRunner
Getting provenance data
|0%                          50%                         100%|
 ============================================================
2019-01-29 14:35:56 INFO: Time 0:00:00.004416 taken by PlacementsProvenanceGatherer
Getting Router Provenance
|0%                          50%                         100%|
 ============================================================
2019-01-29 14:35:56 INFO: Time 0:00:00.084891 taken by RouterProvenanceGatherer
Getting profile data
|0%                          50%                         100%|
 ============================================================
2019-01-29 14:35:56 INFO: Time 0:00:00.004141 taken by ProfileDataGatherer
at time 0





   X
    X
  XXX





at time 1






  X X
   XX
   X




at time 2






    X
  X X
   XX




at time 3
```

```






   X
    XX
   XX




at time 4






    X
     X
   XXX




at time 5







   X X
    XX




at time 6







     X
   X X




at time 7






```

```

    X
     XX




at time 8







     X
      X




at time 9
     X







    X X




at time 10
     XX







      X




at time 11
     XX







     X



```

```

at time 12
     XXX








      X




at time 13
      XX
      X













at time 14
     X X
      XX













at time 15
       XX
      XX













at time 16
        X
      XXX


```

```
at time 17
      X X
       XX
       X

at time 18
        X
      X X
       XX

at time 19
       X
        XX
       XX

at time 20
        X
         X
       XXX
```

```
at time 21

       X X
        XX
        X

at time 22

         X
       X X
        XX

at time 23

        X
X        X
        XX

at time 24

         X
X
X       XX

at time 25
```

```
X       X
X        X
         X
at time 26
X
X       X
X        X
at time 27
         X
XX
X        X
at time 28
X
 X
XX       X
at time 29
 X       X
XX
X
```

```





at time 30



 X
 X       X
XX







at time 31



X
 XX
XX







at time 32



 X
  X
XXX







at time 33




X X
 XX
 X






at time 34
```

```




  X
X X
 XX






at time 35




 X
  XX
 XX






at time 36




  X
   X
 XXX






at time 37





 X X
  XX
  X





at time 38






   X
```

```
 X X
  XX





at time 39





  X
   XX
  XX





at time 40





   X
    X
  XXX





at time 41






  X X
   XX
   X




at time 42






    X
  X X
   XX



```

```
at time 43
   X
    XX
   XX

at time 44
    X
     X
   XXX

at time 45
   X X
    XX

at time 46
     X
   X X

at time 47
```

```



    X
     XX




at time 48







     X
      X




at time 49
     X







    X X





```
